# Frequency distribution of cytokine and associated transcription factor single nucleotide polymorphisms in Zimbabweans: Impact on schistosome infection and cytokine levels

**DOI:** 10.1371/journal.pntd.0010536

**Published:** 2022-06-27

**Authors:** Andrew John Hanton, Fiona Scott, Katharina Stenzel, Norman Nausch, Grace Zdesenko, Takafira Mduluza, Francisca Mutapi

**Affiliations:** 1 Institute of Immunology & Infection Research, University of Edinburgh, Ashworth Laboratories, Edinburgh, United Kingdom; 2 NIHR Global Health Research Unit Tackling Infections to Benefit Africa (TIBA), University of Edinburgh, Ashworth Laboratories, Edinburgh, United Kingdom; 3 Department of Biochemistry, University of Zimbabwe, Harare, Zimbabwe; Centers for Disease Control and Prevention, UNITED STATES

## Abstract

Cytokines mediate T-helper (T_H_) responses that are crucial for determining the course of infection and disease. The expression of cytokines is regulated by transcription factors (TFs). Here we present the frequencies of single nucleotide polymorphisms (SNPs) in cytokine and TF genes in a Zimbabwean population, and further relate SNPs to susceptibility to schistosomiasis and cytokine levels. Individuals (N = 850) were genotyped for SNPs across the cytokines *IL4*, *IL10*, *IL13*, *IL33*, and *IFNG*, and their TFs *STAT4*, *STAT5A/B*, *STAT6*, *GATA3*, *FOXP3*, and *TBX21* to determine allele frequencies. Circulatory levels of systemic and parasite-specific IL-4, IL-5, IL-10, IL-13, and IFNγ were quantified via enzyme-linked immunosorbent assay. *Schistosoma haematobium* infection was determined by enumerating parasite eggs excreted in urine by microscopy. SNP allele frequencies were related to infection status by case-control analysis and logistic regression, and egg burdens and systemic and parasite-specific cytokine levels by analysis of variance and linear regression. Novel findings were i) *IL4* rs2070874*T’s association with protection from schistosomiasis, as carriage of ≥1 allele gave an odds ratio of infection of 0.597 (95% CIs, 0.421–0.848, p = 0.0021) and *IFNG* rs2069727*G’s association with susceptibility to schistosomiasis as carriage of ≥1 allele gave an odds ratio of infection of 1.692 (1.229–2.33, p = 0.0013). Neither *IL4* rs2070874*T nor *IFNG* rs2069727*G were significantly associated with cytokine levels. This study found T_H_2-upregulating SNPs were more frequent among the Zimbabwean sample compared to African and European populations, highlighting the value of immunogenetic studies of African populations in the context of infectious diseases and other conditions, including allergic and atopic disease. In addition, the identification of novel infection-associated alleles in both T_H_1- and T_H_2-associated genes highlights the role of both in regulating and controlling responses to *Schistosoma*.

## Author summary

Cytokines are known to modulate the course of infections and immune pathologies, the production of which is partly under genetic control. Genetic variation, such as single nucleotide polymorphisms (SNPs), are therefore known to influence phenotypes in infectious diseases. This study sought to understand how SNPs in genes encoding cytokines and associated transcription factors influence susceptibility and immune responses to schistosomiasis. Here, we report a range of SNPs upregulating immune responses being highly frequent among our Zimbabwean sample, and in addition identify two novel infection-associated loci. Firstly, the *IL4* SNP rs2070874 T allele was identified as protective, while the *IFNG* SNP rs2069727 G allele was identified as a risk allele. Given the paucity of genetic studies focussing on African populations and neglected tropical diseases, our study provides a valuable contribution to our knowledge of the genetic control of schistosomiasis susceptibility with these findings. In addition, we highlight the role of genetics in modulating balance in immune responses and emphasise that further research focussing on African populations is required on this subject in order to improve our understanding of genetics, immune responses, and neglected diseases.

## Introduction

The biological effects of the immune system are partly mediated by the expression of cytokines, which have a strong influence on the type and strength of immune responses. Although these cytokines can be produced by an array of immune cells, their predominant source is T helper (T_H_) cells, particularly CD4^+^ T_H_ cells. These cells have been largely divided into T_H_1, T_H_2, T_H_17 and T-regulatory (Treg) cell types, each characterised by the cytokines they produce. T_H_1 CD4^+^ T cells produce the key T_H_1 cytokines interleukin-2 (IL-2), interferon-γ (IFNγ) and tumor necrosis factor-α, and this response is widely implicated in bacterial and viral infections, while T_H_2 CD4^+^ T cell produce key T_H_2 cytokines IL-4, IL-5 and IL-13, being the key response in parasite infections and allergic reactions [1]. T_H_17 CD4^+^ produce IL-17, while Treg cells produce the regulatory and anti-inflammatory cytokine IL-10 [1]. The balance between these cytokines determines the phenotype of an immune response, subsequent immunopathology, and the eventual clearance or persistence of infection. The production of these cytokines is partly under genetic control via transcription factors (TFs), and changes in individual nucleotides coding for these cytokines or TFs (single nucleotide polymorphisms–SNPs) can significantly alter cytokines and their expression and therefore the nature of the immune response. Both T_H_1 and T_H_2 responses have been implicated in helminth infections in humans, as the balance between these two immune responses has been found to control the development of immunopathological responses [2,3].

Human studies and mouse models have found that the development of fibrotic and granulomatous responses following *Schistosoma* infection is primarily driven by T_H_2 cytokines, with particular emphasis on the roles of IL-5 and IL-13 in driving immunopathology in response to parasite egg deposition [4,5]. Early mouse studies of *S*. *mansoni* infection demonstrated that blockade of the T_H_2 response, either through exogenous administration of IL-12 or knockout of IL-4Rα, inhibits the development of granulomatous and fibrotic responses to schistosomes [6,7]. Conversely, T_H_1 cytokines have been shown to limit immunopathology through a negative feedback loop with T_H_2 responses; for example, high IFNγ production has been found to correlate with reductions in liver fibrosis in mice [5,8,9]. However, studies of *S*. *mansoni* infections in mice lacking the IFNγ receptor found reductions in granuloma size and hastened progression to chronic immune responses to parasites [10]. Therefore, the dynamic between T_H_1 and T_H_2 cytokines in schistosome infection seems critical to directing the typical immunological response, and disruption to either arm may result in abnormal immune responses to infection. In addition to mediating the immune response to *Schistosoma*, our studies have shown that the balance between T_H_1 and T_H_2 responses is of critical importance in the development of protective immunity against schistosomiasis [3,11,12]. Examining how this balance is regulated at the genetic level is highly relevant to understanding how responses, susceptibility to, and resistance to schistosomiasis are biologically mediated.

Host genetics have been implicated in susceptibility human schistosome infection, with the genes localised on chromosome 5 in the region 5q31-q33 having been shown to have key roles [13]. This region carries genes encoding T_H_2 cytokines IL-4, IL-5, and IL-13 [14]. Genetic studies have revealed associations between SNPs in genes encoding cytokines and TFs and susceptibility to schistosomiasis, including in *STAT6*, *IL4*, *IL5*, *IL10*, and *IL13* [15–21]. Recently, Choto and colleagues identified an association between *IL13* rs1800925 and elevated IL-13 concentrations in schistosome-uninfected but not schistosome-infected individuals in Zimbabwe [22]. In addition, Marume and colleagues identified an association between *IL10* SNP rs1800871, protection from *S*. *haematobium* infection and lower IL-10 production [23]. Genetic variation within cytokine and TF genes that are associated with schistosomiasis are often also associated with perturbation to the T_H_1/T_H_2 balance and the expression of cytokines involved in responding to infection. Nonetheless, to date there have been no comprehensive studies documenting the frequency of cytokine- and TF-associated SNPs and their relationship to helminth infection and cytokine levels in an African population, and there is a paucity of genetic research focussing on individuals of African ancestry and neglected tropical diseases [24]. Thus, the aim of this study was to genotype SNPs in cytokine markers of T-helper responses and associated TFs and to relate these to levels of *S*. *haematobium* infection and corresponding cytokines and having done so we identified a novel protective allele in the *IL4* gene (rs2070874*T) and a novel risk allele in the *IFNG* gene (rs2069727*G).

## Methods

### Ethics statement

Ethical approval was granted by the Medical Research Council of Zimbabwe (MRCZ/A/1408) and the University of Zimbabwe Institutional Review Board. The Provincial Medical Director granted local permission. Community members were informed of the study aims and procedures in their local language (Shona), and compliant participants provided written consent or assent from a parent/guardian if aged <18-years-old.

### Study area and participants

This work is part of a larger study characterising the nature and development of schistosome-specific immunity in human populations, with field work conducted from 2008–2010. Participants were recruited from two villages (Magaya and Chipinda) in Murewa District (17°38′49″S 31°46′39″E), Mashonaland East Province, Zimbabwe where *S*. *haematobium* is endemic. The eligibility criteria for this study were as follows: participants had to i) be life-long residents of the area, ii) provide a minimum of two urine and two stool samples on consecutive days and, iii) be negative for *S*. *mansoni*, soil-transmitted helminthiases (STH), malaria and human immunodeficiency virus (HIV). Following the application of inclusion criteria, 850 individuals were recruited to participate in this study. Subsequently, 23 individuals were excluded from parasitological analyses due to missing or incomplete *S*. *haematobium* egg counts, though were included in calculations of genotype and allele frequencies within the study population. The age range of participants was from three-years-old to 86-years-old, and the median age was 12-years old. Participants were 43.88% male and 56.12% female.

### Parasitology and sample collection

*S*. *haematobium*, *S*. *mansoni* and STH eggs were quantified from a minimum of two urine and stool samples, as previously described [3]. Mean *S*. *haematobium* infection intensity was determined by urine microscopy from at least two urine samples provided on consecutive days. While more sensitive tests exist for the detection of lower intensity and prepatent *Schistosoma* infections, such as nucleic acid-based tests or immunological assays, the cost associated with these, given the sample size and field location, was prohibitive [25,26]. 5ml of venous blood was collected from which a drop was used for blood smear microscopic detection of *Plasmodium* spp and 1ml was stored for genotyping studies. The rest of the blood was processed as previously described to extract serum for quantifying cytokine levels and malaria and HIV serodiagnosis [3]. Malaria status was confirmed using Paracheck rapid tests (Orchid Biomedical Systems) and HIV was detected by DoubleCheckGold HIV1&2 Whole Blood test (Orgenics), with positive cases confirmed using Determine HIV1/2 Ag/Ag Combo (InvernessMedical).

### Genotyping

We selected signature cytokines and their associated TFs and conducted a literature search to identify published SNPs in their genes. Candidate SNPs were identified via literature search for the following genes: *IL4*, *IL10*, *IL13*, *IL33*, *IFNG*, *STAT4*, *STAT5A*, *STAT5B*, *STAT6*, *GATA3*, *FOXP3*, and *TBX21*. SNPs were excluded if they had previously been reported to have a minor allele frequency of <0.1 in the Yoruba (West African) population as insufficient allele frequencies would prevent the sufficient statistical power to detect rare effects. Furthermore, those with a recorded association with allergy, asthma, and altered immune system function including effects on cytokine or antibody production were studied further. This resulted in 35 SNPs being selected for this study. SNPs are referred to throughout using the SNP ID registered on the National Center for Biotechnology Information’s (NCBI) SNP database. Genomic DNA was extracted from blood samples and subject to targeted genotyping by sequencing of 35 SNPs, performed by LGC Genomics (Hoddesdon, UK).

### Serology

Both systemic and parasite-specific (antigen-stimulated) IL-4, IL-5, IL-13, IL-10, and IFNγ concentrations were measured by enzyme linked immunosorbent assay (ELISA). A random subgroup of participants (N = 233) resident in Magaya were selected for serological studies and were 48.91% male and 51.09% female. The median age was 12-years-old and the range of ages in this group was from three-years-old to 80-years-old. Both infected and uninfected individuals were included in this analysis. Systemic cytokine levels were determined in duplicate in sera by capture ELISA, as previously described [11]. Parasite-specific cytokines were also measured in duplicate by ELISA from supernatants collected from whole blood cultures stimulated for 48 hours at 37°C with *S*. *haematobium* soluble egg antigen (SEA) (N = 233), cercarial antigen preparation (CAP) (N = 67), or whole worm homogenate (WWH) (N = 233) as previously described [3]. Briefly, sera (systemic cytokines) or blood culture supernatants (parasite-specific cytokines) were added in duplicate to 96-well plates coated with 1ug/ml capture antibody for IL-4, IL-5, IL-13, IL-10 or IFN-γ (BD Biosciences) and incubated overnight at 4°C. Subsequently, 0.5μg/ml (IFNγ only) or 1μg/ml biotinylated detection antibody and was added for two hours at 37°C before streptavidin-horse radish peroxidase for two hours at 37°C. Lastly, 3,3’-5,5’-tetramethylbenzidine substrate was added and developed for five minutes. Samples were then analysed with spectrophotometry at 450nm and compared to a standard curve for each cytokine for quantification.

### Statistical analysis

All statistical analyses were performed using SPSS Statistics Version 25 unless otherwise stated. Infection intensities (egg counts, measured as eggs/10ml urine) and cytokine concentrations were log_10_(x+1) and square-root(x+1) transformed, respectively, in statistical analyses to meet the assumptions of parametric analysis. All figures were produced in GraphPad Prism 8 Version 9.1.0 for Windows (GraphPad Software, San Diego, California USA, www.graphpad.com), unless otherwise stated. 95% confidence intervals (CIs) were calculated for frequencies and proportions using exact binomial tests. Individuals included in analyses were not case-matched, but potential confounders were accounted for in statistical models. These included participant village, age, and sex when analysing infection status/intensity, and participant infection intensity, age, and sex when analysing cytokine concentrations. Controlling for village when analysing cytokine levels was not necessary as cytokine quantification was performed only on individuals residing in Magaya.

### Allele frequency analysis

Minor allele frequencies (MAFs) among individuals of African and European ancestry for each SNP were obtained from the NCBI ALFA database [27] and Pearson’s Chi-Square tests were performed to compare these frequencies with those of the study population. LD between SNPs was analysed using Haploview Version 2 and PLINK Version 1.9 [28,29]. Haplotype blocks of SNPs in strong LD were defined as one or more pairs of SNPs where the 95% CIs of the D’ value between them has a lower limit ≥0.7 and an upper limit ≥0.98 [30]. SNPs that significantly (p < 0.0001) deviated from the Hardy Weinberg Equilibrium (HWE) were excluded. A total of 54 individuals were excluded on the basis of >50% missing genotypes or missing parasitological data, therefore 796 individuals were included in this analysis.

### Relating genotype of single cytokines to S. haematobium infection status and cytokine levels

Pearson’s Chi-Square tests were performed using Haploview 2 to test for significant differences in the frequencies of alleles and haplotypes between schistosome-positive and schistosome-negative individuals. Binary logistic regression was conducted using the genotype of SNPs as predictors of infection while controlling for participant sex, village, and age. This analysis was performed using genotypic (AA vs Aa, AA vs aa), dominant (AA vs Aa + aa), and recessive (AA + Aa vs aa) genetic models to further examine these associations (where A = reference allele, a = minor allele) [31]. Individual SNPs were also related to both systemic and parasite-specific cytokine (IL-4, IL-5, IL-10, IL-13, IFNγ) concentrations. Transformed cytokine concentrations were subject to analysis of variance (ANOVA) (sequential sums of squares) and the effect of each SNP measured following adjustment for confounding variables of sex, age, and transformed infection intensity. Following this, significant overall effects were further analysed by post-hoc pairwise comparisons, adjusted by Bonferroni correction to account for multiple testing. Some relationships could not be reliably tested due to small sample sizes (N < 10) arising from infrequent genotypes, and thus were not included.

### Relating SNP principal components to infection and cytokine levels

Genotypes of all SNPs were subject to PCA. PCs with an eigenvalue >1 and factor loadings ≥0.5 or ≤-0.5, or the highest loading score for a SNP if all were ≤0.5 or ≥-0.5, were included in analyses. Scores for each PC for each individual were extracted using the regression method. Binary logistic regression and multiple linear regression were utilised to predict infection status and intensity, respectively, adjusting for participant village, sex, and age before PC scores were entered stepwise as predictors. Cytokine concentrations were also related to PCs through multiple linear regression. Systemic and parasite-specific IL-4, IL-5, IL-10, IL-13 and IFNγ concentrations were entered as dependent variables into regression models including participant sex, age, and transformed infection intensity as confounders, before PCs were entered stepwise to identify significant relationships.

## Results

### S. haematobium epidemiology

**Fig 1:**
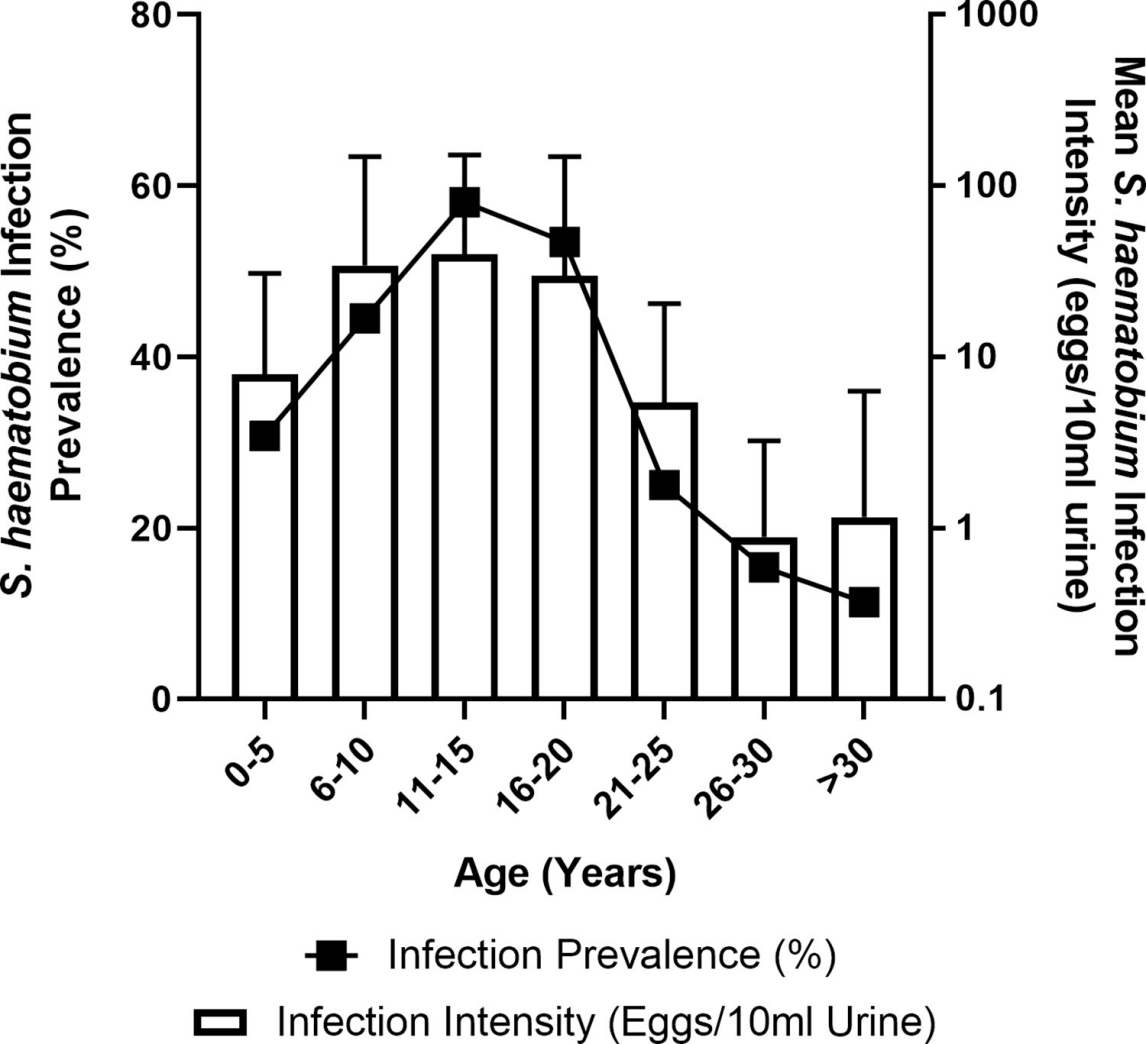
S. haematobium epidemiology across age groups. The epidemiological data indicated that children aged 11 to 15-years-old experienced both the highest S. haematobium prevalence (%) and intensity (eggs/10ml urine), and that the lowest levels were experienced by individuals 26-years-old and older. Error bars indicate SD.

The prevalence of *S*. *haematobium* infection within the study population was 44.498%, and the mean infection intensity was 29.033 eggs/10ml urine (+/- SD 100.646) (*S1 Appendix*). Infection prevalence was highest among 11-15-year-olds (58.065%) and lowest in individuals >30-years-old (11.321%) (*Fig 1*). Similarly, mean infection intensity was highest among 11-15-year-olds (39.691 eggs/10ml urine +/- SD 111.728) and lowest among individuals 26-30-years-old (0.885 eggs/10ml urine, +/- SD 2.347) (*Fig 1*). Additionally, *S*. *haematobium* infection was more prevalent among males (51.648% infected) compared to females (38.745% infected), and mean infection intensity was also higher among males (42.757 eggs/10ml urine, +/- SD 124.862) compared to females (18.162 eggs/10ml urine, +/- SD 74.865). Infection prevalence was higher in Magaya (51.338%) compared to Chipinda (37.740%), however mean infection intensity was lower in Magaya (25.932 eggs/10ml urine, +/- SD 82.628) compared to Chipinda (32.098 eggs/10ml urine, +/- SD 115.747).

### Genetic case-control analysis

**Fig 2:**
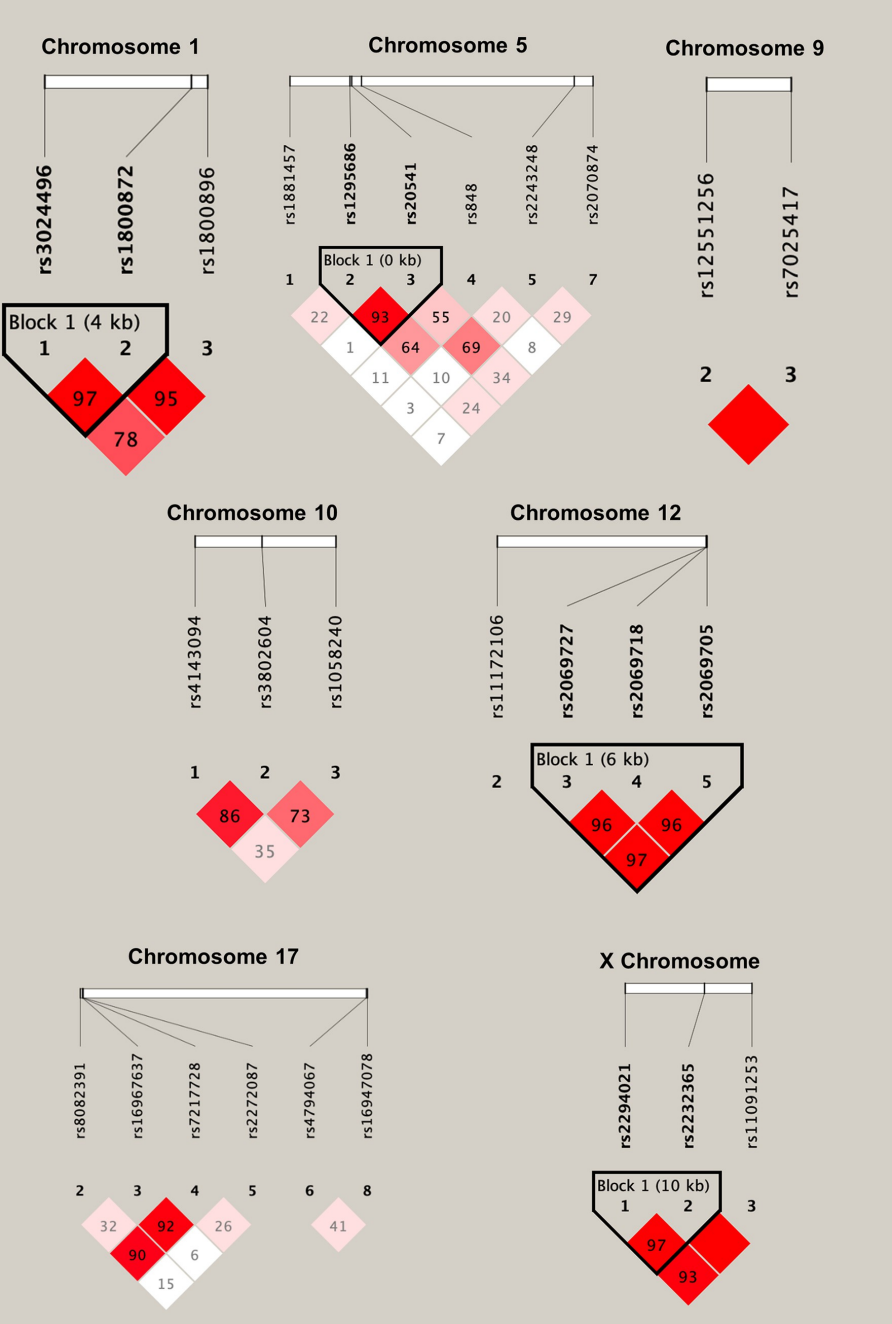
Linkage disequilibrium blocks for SNPs on chromosomes 1, 5, 9, 10, 12, 17 and X. The values within each box indicate the D’ value associated with each pair of SNPs. Blocks indicate groups of two or more SNPs which were found to be in strong LD. Strongly red blocks represent higher degrees of LD (i.e., a higher D’), and whiter blocks represent lower degrees of LD. These results show four haplotype blocks between SNPs on chromosomes 1 (IL10 rs3024496 and IL10 rs1800872), 5 (IL13 rs1295686 and IL13 rs20541), 12 (IFNG rs2069727, IFNG rs2069718 and IFNG rs2069705), and X (FOXP3 rs2294021 and FOXP3 rs2232365), where there exists strong evidence of co-inheritance of SNPs. Plots produced using Haploview 2.

Genotype frequencies (*S2 Appendix)* were used to calculate the MAF of each SNP within the study population (*Table 1*). The mean genotyping completion rate was 97.031%. When comparing MAFs within this sample to MAFs reported by NCBI’s Allele Frequency Aggregator (ALFA) within African and European populations, 23/35 (65.71%) SNPs were significantly different between the Zimbabwean sample and African populations, and 32/35 (91.43%) were significantly different between the Zimbabwean sample and Europeans (*Table 1 and S3 Appendix*). Seven SNPs significantly diverged from the HWE within the study population (*STAT4* rs7574865, *STAT4* rs7582694, *IL4* rs2243250, *IL33* rs928413, *STAT6* rs324015, *STAT5B* rs9900213, and *TBX21* rs11079788) and were excluded from case-control allele frequency analyses. Four haplotype blocks of SNPs in strong linkage disequilibrium (LD) were identified among SNPs in the *IL10* (rs3024496, rs1800872), *IL13* (rs1295686, rs20541), *IFNG* (rs2069727, rs2069718, rs2069705), and *FOXP3* (rs2294021, rs2232365) genes (*Table 2 and Fig 2 and S4 Appendix*). Allele frequencies were then compared in a case-control analysis between schistosome-infected (case) and schistosome-uninfected (control) individuals (*Table 3*). The *IL4* SNP rs2070874 minor allele T (rs2070874*T) was found to have a significantly lower frequency in cases (0.447, 95% CIs: 0.44–0.515) compared to controls (0.554, 95% CIs: 0.521–0.587) (χ^2^ = 9.314, p = 0.0023). Secondly, the *IFNG* SNP rs2069727 minor allele G (rs2069727*G) was found to have a significantly higher frequency in cases (0.172, 95% CIs: 0.145–0.202) compared to controls (0.13, 95% CIs: 0.109–0.154) (χ^2^ = 5.532, p = 0.0187). Lastly, haplotype block 3, consisting of the *IFNG* SNPs rs2069727, rs2069718, and rs2069705 had a frequency of the haplotype GGT of 0.168 (95% CIs: 0.152–0.185) in cases, and 0.128 (95% CIs: 0.116–0.142) in controls (χ^2^ = 4.862, p = 0.0275) (*Table 4*), though this effect was weaker than that seen for *IFNG* rs2069727*G alone.

**Table 1:** Minor allele frequencies of SNPs in Zimbabwean sample, and Chi-square comparative analysis of frequencies between Zimbabwean sample and African and European populations.

Chromosome	Gene	Gene ID	Position	SNP ID	Nucleotide Change	Reported SNP Phenotype	Zimbabwe MAF (95% CIs)	African MAF			European MAF		
								ALFA MAF (95% CIs)	Χ^2^	p	ALFA MAF (95% CIs)	Χ^2^	p
1	*IL10*	3586	206768519	rs3024496	T>C	Decreased IL-10 [32] and IgE [19]	0.462 (0.438–0.487)	**0.399 (0.386–0.412)**	**11.865**	**0.00057**	0.481 (0.477–0.485)	1.231	0.27
			206773062	rs1800872	A>C	Decreased IL-10 [33] and IgE [19,34]	0.606 (0.582–0.63)	0.588 (0.572–0.604)	0.922	0.34	**0.773 (0.759–0.786)**	**99.064**	**<0.0001**
			206773552	rs1800896	A>G	Increased IL-10 [35,36]	0.306 (0.285–0.33)	0.333 (0.317–0.349)	2.203	0.14	**0.481 (0.478–0.484)**	**101.122**	**<0.0001**
2	*STAT4*	6775	191032814	rs925847	C>T	Reduced eczema [37]	0.454 (0.43–0.478)	**0.408 (0.395–0.421)**	**6.205**	**0.013**	**0.275 (0.272–0.278)**	**131.161**	**<0.0001**
			191099907	rs7574865	T>G	Reduced autoimmune disease risk [38–41]	0.872 (0.855–0.888)	0.854 (0.845–0.863)	1.846	0.17	**0.775 (0.773–0.777)**	**44.098**	**<0.0001**
			191105394	rs7582694	C>G	Reduced autoimmune disease risk [42,43]	0.787 (0.766–0.806)	0.83 (0.73–0.9)	0.815	0.37	0.774 (0.756–0.792)	0.074	0.79
5	*IL13*	3596	132656717	rs1881457	A>C	Decreased IL-13 [44] and allergy [45]	0.245 (0.225–0.267)	**0.199 (0.184–0.214)**	**8.191**	**0.0042**	**0.199 (0.196–0.202)**	**11.075**	**0.00087**
			132660151	rs1295686	A>G	Decreased asthma [46,47] and IgE [47]	0.255 (0.234–0.277)	**0.375 (0.363–0.387)**	**44.871**	**<0.0001**	**0.796 (0.794–0.798)0.807**	**1441.949**	**<0.0001**
			132660272	rs20541	A>G	Higher *S*. *japonicum* risk [48] risk, reduced allergy [49–53] reduced IgE [50]	0.765 (0.744–0.785)	**0.807 (0.798–0.816)**	**8.305**	**0.0040**	**0.802 (0.8–0.804)**	**7.128**	**0.0076**
			132660808	rs848	T>G	Increased psoriasis [54] and asthma severity [55]	0.462 (0.438–0.487)	0.5 (0.482–0.518)	3.664	0.056	**0.804 (0.801–0.807)**	**584.157**	**<0.0001**
	*IL4*	3565	132672952	rs2243248	T>G	Reduced asthma [56–58] and allergy [59]	0.203 (0.183–0.223)	**0.154 (0.145–0.163)**	**30.981**	**<0.0001**	**0.069 (0.067–0.071)**	**325.991**	**<0.0001**
			132673462	rs2243250	C>T	Higher *S*. *haematobium* risk [21], increased IL-4 [60–62] and IgE [63]	0.772 (0.75–0.792)	**0.642 (0.625–0.659)**	**48.426**	**<0.0001**	**0.144 (0.142–0.146)**	**2490.361**	**<0.0001**
			132674018	rs2070874	C>T	Increased asthma [64–68] IL-4 and IgE [68]	0.542 (0.517–0.566)	**0.397 (0.385–0.409)**	**63.044**	**<0.0001**	**0.143 (0.141–0.145)**	**1041.882**	**<0.0001**
9	*IL33*	90865	6213387	rs928413	G>A	Reduced allergy [69,70] and asthma [71]	0.393 (0.369–0.418)	**0.513 (0.498–0.528)**	**37.651**	**<0.0001**	**0.749 (0.744–0.754)**	**501.834**	**<0.0001**
			6231239	rs12551256	A>G	Reduced asthma [72,73]	0.117 (0.102–0.133)	**0.216 (0.206–0.227)**	**44.027**	**<0.0001**	**0.466 (0.463–0.469)**	**408.294**	**<0.0001**
			6240084	rs7025417	T>C	Decreased IL-33 [74,75]	0.182 (0.164–0.202)	0.196 (0.183–0.209)	2.684	0.10	**0.223 (0.22–0.227)**	**7.879**	**0.0050**
10	*GATA3*	2625	8047173	rs4143094	T>G	Increased colorectal cancer risk [76] and increased allergy [77]	0.506 (0.481–0.53)	0.537 (0.514–0.559)	2.139	0.14	**0.746 (0.743–0.749)**	**243.209**	**<0.0001**
			8060309	rs3802604	G>A	Reduced breast cancer risk [78,79]	0.218 (0.199–0.239)	**0.253 (0.238–0.268)**	**4.308**	**0.038**	**0.637 (0.634–0.64)**	**615.58**	**<0.0001**
			8074635	rs1058240	G>A	Increased GATA3 expression [80] and reduced allergy [81]	0.818 (0.798–0.836)	**0.763 (0.753–0.773)**	**12.718**	**0.00036**	0.81 (0.808–0.812)	0.368	0.54
12	*STAT6*	6778	68154443	rs324015	A>G	Reduced atopic asthma risk [81] and reduced IgE [82]	0.816 (0.796–0.834)	0.788 (0.774–0.802)	3.138	0.077	**0.762 (0.759–0.764)**	**13.119**	**0.00029**
			68156382	rs11172106	C>G	Increased cord blood IgE [83]	0.318 (0.295–0.34)	0.32 (0.219–0.429)	0.00056	0.98	**0.451 (0.429–0.472)**	**21.917**	**<0.0001**
	*IFNG*	3458	68161231	rs2069727	A>G	Reduced IFNγ [84] and sex-dependent asthma risk [85]	0.153 (0.136–0.171)	**0.212 (0.199–0.225)**	**14.981**	**0.00011**	**0.468 (0.465–0.471)**	**329.253**	**<0.0001**
			57096317	rs2069718	A>G	Reduced tuberculosis susceptibility [83,86,87]	0.361 (0.338–0.385)	**0.409 (0.394–0.425)**	**6.406**	**0.011**	**0.605 (0.602–0.608)**	**201.502**	**<0.0001**
			57119092	rs2069705	C>T	Reduced tuberculosis [88] and increased cutaneous leishmaniasis susceptibility [89]	0.542 (0.517–0.566)	0.568 (0.556–0.58)	2.044	0.15	**0.673 (0.67–0.676)**	**63.898**	**<0.0001**
17	*STAT5B*	6777	42223863	rs9900213	G>T	Higher serum cholesterol [90]	0.663 (0.64–0.686)	**0.552 (0.535–0.569)**	**33.795**	**<0.0001**	**0.164 (0.162–0.166)**	**1476.438**	**<0.0001**
			42246955	rs8082391	C>A	Higher serum cholesterol [91]	0.612 (0.588–0.635)	**0.521 (0.504–0.538)**	**22.511**	**<0.0001**	**0.301 (0.298–0.304)**	**379.185**	**<0.0001**
	*STAT5A*	6776	42294404	rs16967637	C>A	Higher serum cholesterol [90]	0.376 (0.353–0.4)	0.376 (0.341–0.412)	<0.001	0.98	**0.299 (0.285–0.313)**	**18.66**	**<0.0001**
			42295383	rs7217728	T>C	Reduced colon cancer risk [92]	0.703 (0.68–0.723)	**0.587 (0.571–0.603)**	**38.381**	**<0.0001**	**0.301 (0.298–0.304)**	**688.884**	**<0.0001**
			42307544	rs2272087	T>C	Higher serum cholesterol [90]	0.339 (0.316–0.362)	**0.241 (0.209–0.275)**	**17.266**	**<0.0001**	**0.19 (0.182–0.198)**	**104.365**	**<0.0001**
	*TBX21*	30009	47731462	rs4794067	T>C	Reduced TBX21 and IFNγ expression [93,94]	0.147 (0.13–0.165)	**0.197 (0.187–0.207)**	**11.422**	**0.00073**	**0.271 (0.269–0.273)**	**62.649**	**<0.0001**
			47743357	rs11079788	C>T	Increased CD4^+^ T cell activation [95]	0.049 (0.039–0.06)	**0.236 (0.213–0.26)**	**130.616**	**<0.0001**	**0.219 (0.194–0.246)**	**107.850**	**<0.0001**
			47748134	rs16947078	A>G	Increased asthma risk [96]	0.161 (0.144–0.18)	**0.201 (0.188–0.214)**	**7.081**	**0.0078**	**0.221 (0.218–0.224)**	**17.279**	**<0.0001**
X	*FOXP3*	50943	49249149	rs2294021	T>C	Increased FOXP3 expression [96,97] and increased Tregs and IL-10 [98]	0.288 (0.266–0.311)	**0.42 (0.406–0.434)**	**50.718**	**<0.0001**	**0.58 (0.576–0.584)**	**283.831**	**<0.0001**
			49259429	rs2232365	A>G	Higher FOXP3 expression [99]	0.751 (0.73–0.772)	0.787 (0.763–0.81)	3.742	0.053	**0.598 (0.582–0.614)**	**67.189**	**<0.0001**
			49265564	rs11091253	C>T	Increased *P*. *falciparum* malaria risk [100]	0.197 (0.178–0.217)	**0.259 (0.235–0.285)**	**10.9**	**0.00096**	**0.0003 (0.000006–0.0015)**	**758.64**	**<0.0001**

**Table 2:** Linkage disequilibrium statistics of haplotype blocks.

Block	Chromosome	Gene	SNP 1 ID	SNP 2 ID	D’ (95% CIs)	r^2^
1	1	*IL10*	rs3024496	rs1800872	0.973 (0.94–0.99)	0.547
2	5	*IL13*	rs1295686	rs20541	0.93 (0.84–0.98)	0.097
3	12	*IFNG*	rs2069727	rs2069718	0.968 (0.92–0.99)	0.307
			rs2069727	rs2069705	0.971 (0.9–1.0)	0.151
			rs2069718	rs2069705	0.969 (0.93–0.99)	0.458
4	X	*FOXP3*	rs2294021	rs2232365	0.971 (0.9–1.0)	0.558

**Table 3:** Case-control analysis of SNP MAFs between schistosome-infected (N = 354) and–uninfected individuals (N = 442).

Chromosome	Gene	SNP ID	Nucleotide Change	Allele	Case:Control Frequency	χ^2^	p
1	*IL10*	rs3024496	T>C	T	0.544:0.554	0.176	0.68
				C	0.456:0.446		
		rs1800872	A>C	A	0.418:0.406	0.233	0.63
				C	0.582:0.594		
		rs1800896	A>G	A	0.718:0.688	1.658	0.20
				G	0.282:0.312		
2	*STAT4*	rs925847	C>T	C	0.565:0.546	0.55	0.46
				T	0.435:0.454		
5	*IL13*	rs1881457	A>C	A	0.763:0.757	0.076	0.78
				C	0.237:0.243		
		rs1295686	A>G	A	0.757:0.757	0.000	0.99
				G	0.243:0.243		
		rs20541	A>G	A	0.237:0.269	2.111	0.15
				G	0.763:0.731		
		rs848	T>G	T	0.549:0.528	0.708	0.40
				G	0.451:0.472		
	*IL4*	rs2243248	T>G	T	0.798:0.8	0.008	0.93
				G	0.202:0.2		
		rs2070874	C>T	C	0.523:0.446	**9.314**	**0.0023**
				T	0.447:0.554		
9	*IL33*	rs12551256	A>G	A	0.884:0.888	0.057	0.81
				G	0.116:0.112		
		rs7025417	T>C	T	0.823:0.819	0.053	0.82
				C	0.177:0.181		
10	*GATA3*	rs4143094	T>G	T	0.532:0.505	1.231	0.27
				G	0.438:0.495		
		rs3802604	G>A	G	0.804:0.781	1.271	0.26
				A	0.196:219		
		rs1058240	G>A	G	0.203:0.192	0.305	0.58
				A	0.797:0.808		
12	*STAT6*	rs11172106	C>G	C	0.709:0.689	0.755	0.38
				G	0.291:0.311		
	*IFNG*	rs2069727	A>G	A	0.828:0.87	**5.532**	**0.019**
				G	0.172:0.13		
		rs2069718	A>G	A	0.641:0.655	0.325	0.57
				G	0.359:0.345		
		rs2069705	C>T	C	0.458:0.484	1.111	0.29
				T	0.542:0.516		
17	*STAT5*	rs8082391	C>A	C	0.403:0.398	0.031	0.86
				A	0.597:0.602		
		rs16967637	C>A	C	0.643:0.611	1.696	0.19
				A	0.357:0.389		
		rs7217728	T>C	T	0.319:0.309	0.197	0.66
				C	0.681:0.691		
		rs2272087	T>C	T	0.606:0.624	0.569	0.45
				C	0.394:0.376		
	*TBX21*	rs4794067	T>C	T	0.866:0.851	0.737	0.39
				C	0.134:0.149		
		rs16947078	A>G	A	0.849:0.839	0.269	0.60
				G	0.151:0.161		
X	*FOXP3*	rs2294021	T>C	T	0.726:0.714	0.206	0.65
				C	0.274:0.286		
		rs2232365	A>G	A	0.271:0.257	0.323	0.57
				G	0.729:0.743		
		rs11091253	C>T	C	0.816:0.792	1.092	0.27
				T	0.184:0.208		

**Table 4:** Case-control analysis of haplotype frequencies between schistosome-infected (N = 354) and–uninfected individuals (N = 442).

Block	Chromo-some	Gene	SNP IDs	4Haplotype	Case:Control Frequency	χ^2^	p
1	1	*IL10*	rs3024496 rs1800872	CC	0.453:0.441	0.231	0.63
				TA	0.414:0.401	0.292	0.59
				TC	0.13:0.153	1.815	0.18
2	2	*IL13*	rs1295686 rs20541	AG	0.525:0.491	1.835	0.18
				AA	0.233:0.266	2.296	0.13
				GG	0.237:0.24	0.018	0.89
3	12	IFNG	rs2069727 rs2069718 rs2069705	AAC	0.448:0.482	1.825	0.18
				AGT	0.184:0.215	2.268	0.13
				AAT	0.189:0.171	0.803	0.37
				GGT	0.168:0.128	**4.862**	**0.028**
4	X	*FOXP3*	rs2294021 rs2232365	TG	0.454:0.46	0.055	0.81
				CG	0.274:0.283	0.111	0.74
				TA	0.272:0.253	0.515	0.47

### Regression analysis of S. haematobium infection

**Fig 3:**
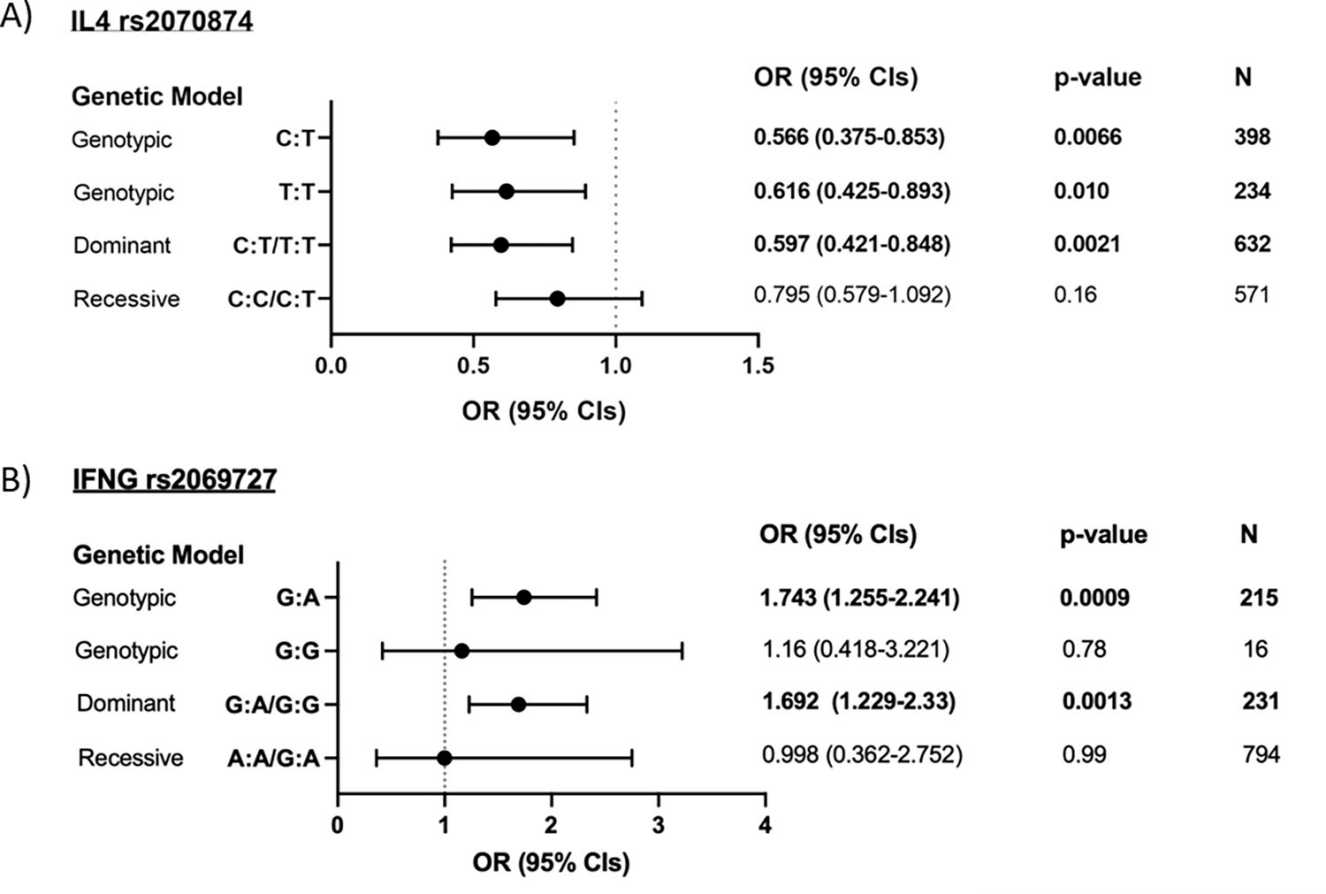
A) Odds ratios of S. haematobium infection between genotypes of SNPs IL4 rs2070874 (N = 805) and B) IFNG rs2069727 (N = 810). Models are adjusted for the confounding variables of participant age, sex and village. These data indicate that the C:T and T:T genotypes and the T allele of IL4 rs2070874 was associated with protection from S. haematobium infection, and that the G:A genotype and the G allele of IFNG rs2069727 was associated with elevated risk of infection with S. haematobium. OR = odds ratio; CIs = confidence intervals. Genotypic and dominant models display ORs relative to the homozygous reference genotype; recessive models display ORs relative to the homozygous variant genotype.

Corroborating the previous analysis, *IL4* rs2070874*T and *IFNG* rs2069727*G were significantly associated with infection in a logistic regression model after adjusting for the confounders of age, sex and village. Individuals with the *IL4* rs2070874 genotypes C:T or T:T had ORs of 0.566 (95% CIs: 0.375–0.853, p = 0.0066) and 0.616 (95% CIs: 0.425–0.893, p = 0.010), respectively, relative to the C:C genotype (*Fig 3A*). Additionally, in a dominant model, individuals carrying at least one copy of *IL4* rs2070874*T had an OR of 0.597 (95% CIs: 0.421–0.848, p = 0.0021) relative to the C:C genotype. Under a recessive model, no significant differences were found when comparing individuals carrying at least one copy of the C allele to those homozygous for the T allele. Secondly, individuals with the *IFNG* rs2069727 genotype G:A had an OR of 1.743 (1.255–2.421, p = 0.0009) relative to individuals with the A:A genotype (*Fig 3B*). Individuals with the G:G genotype were not found to have a significant OR for egg positivity relative to individuals with the A:A genotype, however the G:G genotype had a frequency of 0.019 (N = 16), thus this analysis lacks power. In a dominant model, individuals carrying at least one copy of *IFNG* rs2069727*G had a combined OR of 1.692 (95% CIs: 1.229–2.33, p = 0.0013), relative to individuals with the A:A genotype. In addition, under a recessive model, no significant differences were found.

### Analysis of cytokine production

**Fig 4:**
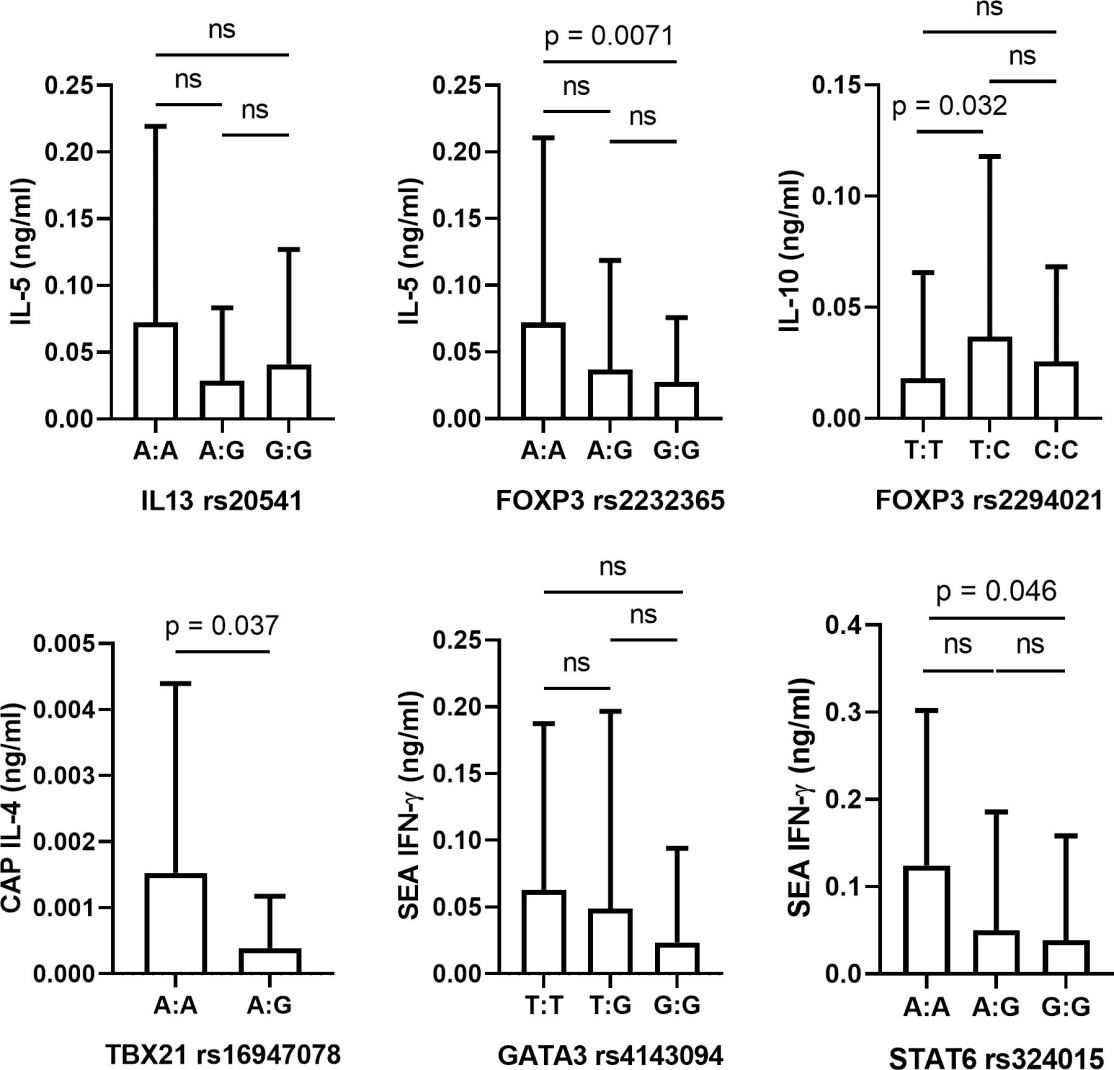
Mean cytokine concentrations between SNP genotypes. Analysis of variance identified relationships between systemic or parasite specific cytokine levels and six SNPs: IL13 rs20541 (systemic IL-5), FOXP3 rs2232365 (systemic IL-5), FOXP3 rs2294021 (systemic IL-10), TBX21 rs16947078 (CAP-specific IL-4), GATA3 rs4143094 (SEA-specific IFNγ), and STAT6 rs324015 (SEA-specific IFNγ). Pairwise p-values are Bonferroni corrected. Error bars indicate SD. CAP = cercariae antigen preparation; SEA = soluble egg antigen).

A subgroup of participants (N = 233) resident in Magaya were further investigated to study cytokine responses. Among this subgroup, the prevalence of *S*. *haematobium* infection was 52.586%, and the mean egg count was 29.997 eggs/10ml urine (+/- SD 87.298). Participants were grouped on the basis of SNP genotype and not infection status, and therefore infection status-dependent effects were not examined. SNPs were investigated to analyse effects on systemic and parasite-specific cytokine concentrations using ANOVA to compare genotypes (*Fig 4 and Table 5*). This indicated six significant relationships between SNPs and cytokine levels following adjustment for sex, age, and infection. Firstly, *IL13* rs20541 was significantly associated with systemic IL-5 concentrations (F = 4.318, p = 0.015), whereby individuals with the A:A genotype had a higher mean IL-5 concentration than individuals with the A:G and G:G genotypes, however these comparisons were not significant following Bonferroni post-hoc analysis. *FOXP3* rs2232365 was also significantly associated with systemic IL-5 concentrations (F = 3.382, p = 0.037), and post-hoc analysis found that individuals with the A:A genotype had a significantly higher mean IL-5 concentration compared to individuals with the G:G genotype (p = 0.0071). Systemic IL-10 was significantly associated with the *FOXP3* SNP rs2294021 (F = 3.315, p = 0.0039), and post-hoc analysis indicated that individuals with the T:T genotype had a significantly lower mean IL-10 concentration compared to individuals with the T:C genotype (p = 0.032) but not those with the C:C genotype. Parasite-specific cytokine levels were also influenced by SNP genotypes. CAP-specific IL-4 was significantly associated with the *TBX21* SNP rs16947078 (F = 4.763, p = 0.037), whereby individuals with the A:A genotype had a significantly higher mean concentration compared to individuals of the A:G genotype (note: CAP-specific IL-4 was not measured in any individuals with the G:G genotype). Additionally, SEA-specific IFNγ was associated with both *GATA3* rs4143094 and *STAT6* rs324015. Firstly, *GATA3* rs4143094 was significantly associated with SEA-specific IFNγ (F = 4.212, p = 0.017), with a trend towards lower mean concentrations associated with the G allele, however post-hoc analysis did not indicate any significant pairwise comparisons between genotypes. Lastly, SEA-specific IFNγ was significantly associated with *STAT6* rs324015 (F = 4.857, p = 0.0092), and post-hoc analyses indicated that individuals with the A:A genotype had a significantly higher mean SEA-specific IFNγ concentration compared to individuals with the G:G genotype (p = 0.046).

**Table 5:** Analysis of variance of mean cytokine concentrations between SNP genotypes.

Cytokine	SNP	Genotype (N)	Mean Concentration (SD) (ng/ml)	F	p-value
IL-5	*IL13* rs20541	A:A (13)	0.7234 (0.1468)	4.3184	**0.015**
		A:G (88)	0.02855 (0.0547)		
		G:G (124)	0.0409 (0.0861)		
	*FOXP3* rs2232365	A:A (39)	0.0720 (0.1384)	3.3824	**0.037**
		A:G (40)	0.0369 (0.0818)		
		G:G (149)	0.0273 (0.0483)		
IL-10	*FOXP3* rs2294021	T:T (147)	0.0180 (0.0475)	3.3152	**0.039**
		T:C (42)	0.0368 (0.0810)		
		C:C (36)	0.0235 (0.06948)		
CAP IL-4	*TBX21* rs16947078	A:A (47)	0.0015 (0.0029)	4.7627	**0.037**
		A:G (19)	0.0004 (0.0008)		
SEA IFNγ	*GATA3* rs4143094	T:T (55)	0.0629 (0.1245)	4.2122	**0.017**
		T:G (117)	0.0487 (0.1478)		
		G:G (50)	0.0229 (0.0700)		
	*STAT6* rs324015	A:A (55)	0.1243 (0.1778)	4.8574	**0.0092**
		A:G (117)	0.04969 (0.1358)		
		G:G (50)	0.0385 (0.1196)		

### Principal component analysis

Principal component analysis (PCA) of the 35 SNPs studied here resulted in the identification of 14 PCs representing 67.52% of total variance (*S5 Appendix*). SNPs were scored 1, 2 or 3 to represent homozygous reference, heterozygous, and homozygous variant genotypes, respectively, and as such increasing PC scores indicate an increasing number of variant alleles. Extracted PC scores were then used as predictors of infection status and intensity in logistic and linear regression models, respectively. Firstly, in a logistic model, results corroborated those previously outlined, as PC6 (representing *IL4* SNPs rs2070874, rs2243259 and rs2243248) was associated with decreased odds of *S*. *haematobium* infection (OR = 0.8026, 95% CIs: 0.6713–0.9595, p = 0.016) (*S6 Appendix)*. In a linear model, no PC was significantly associated with infection intensity (*S6 Appendix*).

**Fig 5:**
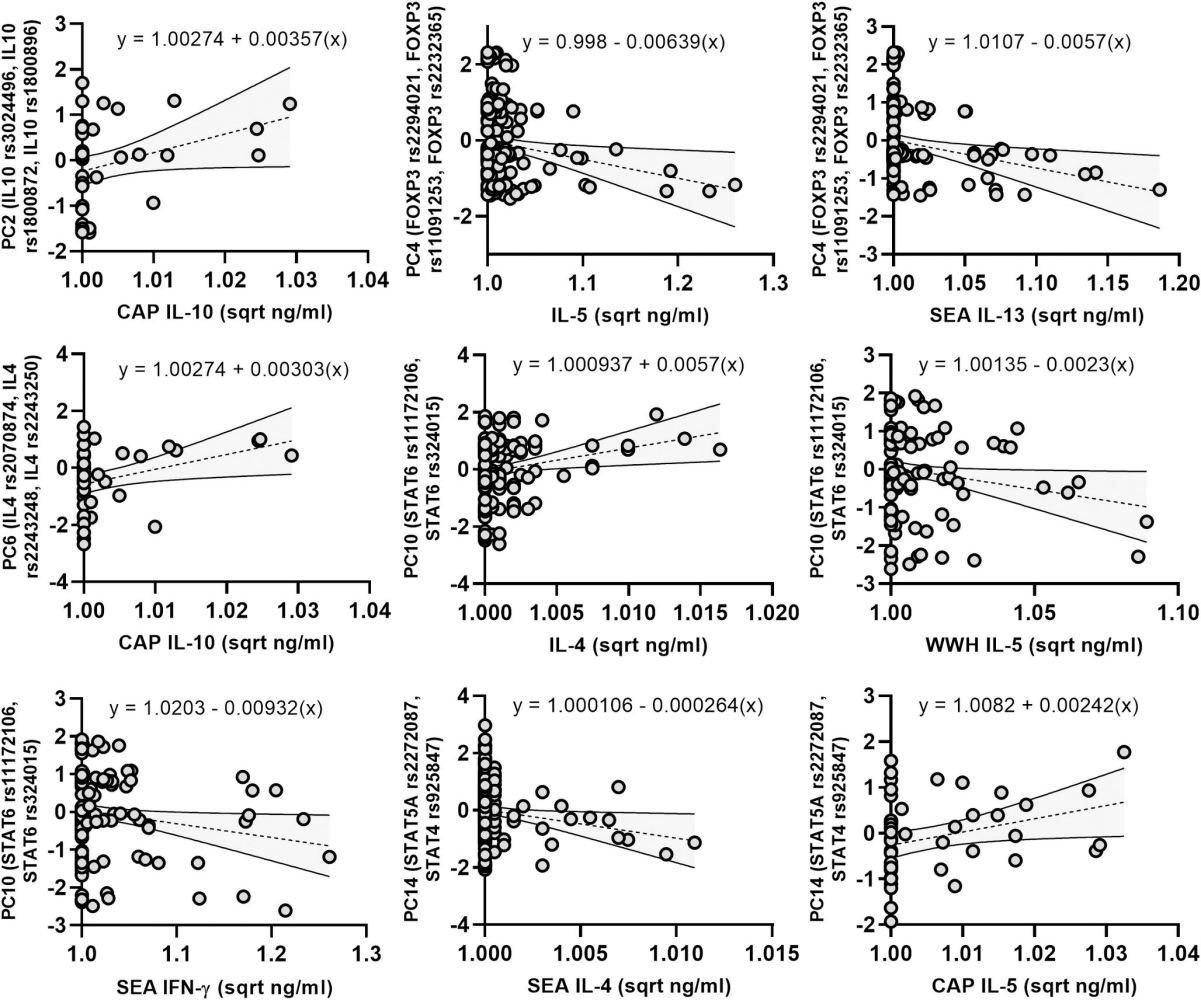
Regression scatterplots of systemic and parasite-specific cytokines against PC scores. X axes show square-root (x+1) transformed values. Dashed line shows regression best line of fit, and shaded area shows 95% confidence intervals of the best line of fit. Regression analysis found significant relationships between systemic and parasite-specific cytokine levels and PC2 (CAP-specific IL-10), PC4 (systemic IL-5, SEA-specific IL-13), PC6 (CAP-specific IL-10), PC10 (systemic IL-4, WWH-specific IL-5, SEA-specific IFNγ) and PC14 (SEA-specific IL-4, CAP-specific IL-5). PC = principal component; CAP = cercariae antigen preparation; SEA = soluble egg antigen; WWH = whole worm homogenate.

Linear regression identified a number of relationships between PCs and cytokine concentrations (*Fig 5 and Table 6*). PC10, representing *STAT6* SNPs rs11172106 and rs324015, was associated with the largest number of cytokine responses–elevated systemic IL-4 concentrations (B = 0.000494 (95% CIs: 0.000115–0.000873) p = 0.011), reduced WWH-specific IL-5 (B = -0.0023 (95% CIs: -0.0047 –-0.0001), p = 0.037) and reduced SEA-specific IFNγ (B = -0.0093 (95% CIs: -0.0179 –-0.0008), p = 0.033). As described in the previous ANOVA, *STAT6* rs324015 was independently associated with reduced SEA-specific IFNγ, although rs11172106 was not, and neither were independently associated with systemic IL-4 or WWH-specific IL-5. PC4, representing *FOXP3* SNPs rs2294021, rs11091253 and rs2232365, and PC14, representing *STAT5A* rs2272087 and *STAT4* rs925847, were each significantly associated with two cytokine responses. Firstly, PC4 was significantly associated with reduced systemic IL-5 (B = -0.0063 (95% CIs: -0.0119 –-0.0008), p = 0.026) and reduced SEA-specific IL-13 (B = -0.0057 (95% CIs: -0.0102 –-0.0014), p = 0.011). *FOXP3* rs2232365 was independently associated with reduced systemic IL-5 concentrations, as previously described, however neither was independently associated with SEA-specific IL-13. PC14 was significantly associated with reduced SEA-specific IL-4 (B = -0.0003 (95% CIs: -0.0005–2.302x10^-5^), p = 0.032) and elevated CAP-specific IL-5 (B = 0.0024 (95% CIs: 8.738x10^-5^–0.0047), p = 0.042), and neither rs2272087 nor rs925847 was independently associated with these cytokine responses. Lastly, CAP-specific IL-10 concentrations were significantly associated with both PC2 (B = 0.0036 (95% CIs: 0.0007–0.0064), p = 0.015) representing *IL10* rs3024496, rs1800872 and rs1800896, and PC6 (B = 0.0030 (95% CIs: 7.586x10^-5^–0.0060), p = 0.045), representing *IL4* SNPs rs2070874, rs2243248 and rs2243250.

**Table 6:** Multiple linear regression of cytokine concentrations and PCs.

PC	SNPs	Cytokine	B-Coefficient (95% CIs)	β-Coefficient (95% CIs)	p-value
2	*IL10* rs3024496 *IL10* rs1800872 *IL10* rs1800896	CAP IL-10	0.00357 (0.000731–0.00641)	0.3500 (0.0717–0.6282)	0.015
4	*FOXP3* rs2294021 *FOXP3* rs11091253 *FOXP3* rs2232365	IL-5	-0.00631 (-0.0119- -0.00076)	-0.1704 (-0.3202- -0.0206)	0.026
		SEA IL-13	-0.00570 (-0.0102- -0.00136)	-0.1975 (-0.3488- -0.0462)	0.011
6	*IL4* rs2070874 *IL4* rs2243248 *IL4* rs2243250	CAP IL-10	0.00303 (7.586x10^-5^–0.00598)	0.2877 (0.0072–0.5682)	0.045
10	*STAT6* rs11172106 *STAT6* rs324015	IL-4	0.000494 (0.000115–0.000873)	0.2005 (0.0467–0.3543)	0.011
		WWH IL-5	-0.0023 (-0.00447- -0.00014)	-0.1613 (-0.3130- -0.0097)	0.037
		SEA IFNg	-0.00932 (-0.0179- -0.000781)	-0.1684 (-0.3228- -0.0141)	0.033
14	*STAT5A* rs2272087 *STAT4* rs925847	SEA IL-4	-0.000264 (-0.000507–2.302x10^-5^)	-0.1664 (-0.3182- -0.0145)	0.032
		CAP IL-5	0.00242 (8.783x10^-5^–0.00474)	0.2960 (0.0108–0.5814)	0.042

## Discussion

Cytokines are crucial for the type of immune response mounted against pathogens, the expression of which is partially controlled by TFs. Here, we analysed the frequency of SNPs in genes encoding cytokine markers of T_H_1, T_H_2 and Treg responses and their associated TFs. We related the presence of these SNPs to the risk of schistosome infection as well as levels of systemic and schistosome-specific cytokines. Our most significant findings were that *IL4* rs2070874*T is significantly associated with a reduction in schistosome infection risk, while *IFNG* rs2069727*G and the haplotype GGT across *IFNG* SNPs rs2069727, rs2069718, rs2069705 were associated with increased schistosome infection risk. For both *IL4* rs2070874*T and *IFNG* rs2069727*G, it is apparent from these results that carriage of one allele is sufficient to elicit the associated phenotype. To our knowledge, this is the first time either SNP has been associated with susceptibility to schistosomiasis. The protective *IL4* rs2070874*T allele had a frequency of 0.542 within the Zimbabwean sample, which is significantly higher than both African and European populations, in which the allele has frequencies of 0.397 and 0.143, respectively. In addition, the risk *IFNG* rs2069727*G allele had a frequency of 0.153 within the Zimbabwean sample, which is significantly lower than both African and European populations, in which the allele has frequencies of 0.212 and 0.468, respectively. Therefore, the protective and risk alleles described here are more and less frequent, respectively, among the study population compared to individuals of both African and European descent.

Of the 35 SNPs under investigation, we found that 65.71% and 91.43% had MAFs that were significantly different between the study sample and African and European populations, respectively. Such high levels of difference between the study population and Europeans are unsurprising. It is equally unsurprising that the study population displayed strong differences to aggregated African populations, as ALFA combines population genetic data across the entire continent of Africa, where there are considerable between- and within-subpopulation genetic differences [101]. By comparing MAFs within the Zimbabwean sample to Africans and Europeans, increased allele frequencies associated with elevated T_H_2 function were found, particularly compared to Europeans. For example, *IL4* rs2243250*T had a frequency of 0.772 within the study sample, compared to 0.642 and 0.144 among Africans and Europeans, respectively, and this allele has previously been associated with the upregulation of T_H_2 responses including increased IL-4 and immunoglobulin-E (IgE) production [60,63]. Additionally, *IL13* rs1295686*G, which has frequencies of 0.225, 0.375 and 0.796 among the Zimbabwean sample, Africans and Europeans, respectively, has been associated with decreased risk of asthma and reduced IgE expression [47].

The rs2070874*T allele was found here to be protective against *S*. *haematobium* and significantly more frequent within the study sample. *IL4* rs2070874 is located within the 5’ untranslated region (UTR) of the *IL4* gene. The 5’ UTR region of genes is associated with controlling translation efficiency through the binding of TFs and RNA polymerase, and the formation of the ribosomal initiation complex [102,103]. This raises the possibility that *IL4* rs2070874*T may influence the translation of *IL4*, and while not observed in this present study, *IL4* rs2070874*T has previously been associated with elevated levels of plasma IL-4 [68]. We also identified a novel association between the *IFNG* gene and schistosomiasis susceptibility, as *IFNG* rs2069727*G was found here to be associated with increased risk of infection. As with *IL4* rs2070874, *IFNG* rs2069727 is not located within a coding region as it is found approximately 500bp downstream of the *IFNG* gene. It is possible that this polymorphism similarly affects the binding of regulatory factors and as such modulates the translation of the *IFNG* gene [103], and though not replicated here, *IFNG* rs2069727*G has previously been associated with altered IFNγ production [84,85]. Further mechanistic investigations are required to fully elucidate the nature of these polymorphisms and their functional impacts, however given that they are both located outside of coding regions, modulations to the regulation of gene expression is a leading hypothesis on the biomolecular consequences of these polymorphisms. In the absence of a mechanistic explanation and without evidence here of associations with cytokine production, it is difficult to draw conclusions on how these SNPs fit into the immunological response to schistosomes and the development of protective immunity. Given each SNP’s identified association with susceptibility to infection, it may be that the biological consequences of these polymorphisms lies in the innate immune response generated following infection. Research by this group and others has found the early immune response to egg deposition relies on both Th1 and Th2 cytokines, including both IL-4 and IFN-γ, as well as cellular elements including alternatively-activated macrophages, monocytes, and innate lymphoid cells, and that these elements both rely on and amplify cytokine responses [3,104–107]. Therefore, it could be that alterations to innate cytokine responses at early stages of infection are influenced by a disruption to the T_H_1/T_H_2 balance arising from these SNPs; however, without further mechanistic evidence, this remains speculative.

Several other SNPs have been identified as influencing risk of schistosome infection, including within the *IL4* gene as Adedokun and colleagues identified an association between *IL4* rs2243250 and increased risk of *S*. *haematobium* in Nigerian children [21]. *IL4* rs2243250 was in violation of HWE in the present study and as such case-control analysis of *S*. *haematobium* infection was not performed for this SNP. Ellis and colleagues conducted a similar genetic association study where they found no association between *IL4* rs2070874 and risk of infection with *S*. *japonicum* in a Chinese population [17]. However, comparability between this study and ours is limited given the differences in population, underlying genetic linkage structures, and *Schistosoma* species. This present study is, to the best of our knowledge, the first genetic association study to examine *IL4* rs2070874 in the context of *S*. *haematobium*. This study is also, to the best of our knowledge, the first to support a genetic association between *IFNG* rs2069727 and infection with *Schistosoma*, and the first to identify an association between schistosomiasis risk and the *IFNG* gene. Due to our finding that *IFNG* rs2069727 is in LD with both *IFNG* rs2069705 and *IFNG* rs2069718, it is difficult to discern the associations with *IFNG* rs2069727 from either of these SNPs. However, given the low r^2^ values between *IFNG* rs2069727, *IFNG* rs2069718, and *IFNG* rs2069705, and the lack of any significant independent association with *IFNG* rs2069705 or *IFNG* rs2069718 alone, the risk allele and associated phenotype is likely to be more closely associated with *IFNG* rs2069727. Previously, *IFNG* rs2069727*G has been associated with elevated IFNγ concentrations [84] and while no association was found here with IFNγ concentrations, it is plausible that an associated disturbance to the T_H_1/T_H_2 balance may underlie the increased risk of schistosomiasis. Therefore, as with *IL4* rs2070874, further examination of the biological effects of these SNPs would prove beneficial. One SNP included in our case-control analysis (*IL13* rs20541) had previously been associated with *S*. *mansoni* and *S*. *japonicum* infection, though these findings were not replicated here [20,48]. There is an apparent lack of reproducibility and generalisability in these associations between populations and *Schistosoma* species, the former of which may be due to differences in LD structures. In addition, it is widely acknowledged that disease susceptibility is mostly the product of whole-genome variation, rather than particularly deleterious or advantageous individual alleles [108]. Therefore, associations being made with individual SNPs or with a limited range of alleles suffer from limited biological relevance. The ability to capture variation across the entire genome would be beneficial in providing a more comprehensive analysis. Our understanding of the complex role of genes in determining susceptibility to schistosome infection is hindered by a lack of GWASs–to date, no published study has performed a GWAS on schistosomiasis in humans. The adoption of such techniques would broaden our knowledge of the role of host genetics in schistosomiasis, other helminthiases, and neglected tropical diseases.

The analysis conducted here identified relationships between SNPs and levels of both systemic and parasite-specific cytokine responses. This included the *FOXP3* SNP rs2232365, which was found to be significantly associated with lower systemic IL-5 both individually and when combined with *FOXP3* SNPs rs11091253 and rs2294021 in PCA-based regression analysis. FOXP3, the master regulatory TF of Treg responses, is responsible also for downregulating T_H_1 and T_H_2 immune responses, and *FOXP3* rs2232365 has been associated with higher *FOXP3* expression [94], potentially underlying the observed decreased IL-5 concentrations. In addition, the *STAT6* SNP rs324015 was associated with reduced SEA-specific IFNγ when comparing genotypes, and this SNP has been previously associated with reduced asthma risk and reduced IgE [81,82], thereby indicating that this polymorphism results in a dampening of T_H_2 responses. Our observation is therefore in accordance with these previous findings. While none of the SNPs identified as being associated with cytokine concentrations were also associated with susceptibility to schistosomiasis, it would be of value to examine whether these SNPs are associated with changes in T_H_2-mediated immunopathology. For example, the *IL13* promoter polymorphism rs1800925 (not studied here) has previously been associated with both elevated IL-13 expression and an increase in liver fibrosis associated with *S*. *japonicum* infection [48]. The observation made here that *STAT6* rs324015 was associated with elevated schistosome egg antigen-specific IFNγ may have implications for early immune responses such as reducing T_H_2-mediated immunopathology following egg deposition, and examination of the immunological consequences of this SNP and others on responses to schistosomiasis would shed further light on the role of host genetics in *S*. *haematobium* infection.

Those SNPs identified here as being significantly associated with *S*. *haematobium* susceptibility were not individually associated with levels of any systemic or parasite-specific cytokines, despite having been associated with expression of their respective cytokines previously [68,84]. A number of reasons exist for this discrepancy, including differences in linkage structures between genes in the study population of this research and that in the study populations of previous research. For example, neither paper previously finding associations between *IL4* rs2070874*T and *IFNG* rs2069727*G and the expression of their respective cytokines studied individuals of African heritage. It is known that individuals of African heritage possess genetic linkage structures significantly different to those of European and other ancestries [109]; therefore, it would not follow that a genetic association in a non-African population would necessarily be replicated in an African population. A weak but significant relationship was identified between the PC representing *IL4* SNPs rs2070874, rs2243248 and rs2243250 and CAP-specific IL-10, however none of these variants were individually associated with these cytokines and therefore it is not possible to deduce which is most likely to be the causative allele of this weak effect. Thus, these results do not provide evidence to hypothesise the underlying mechanism between infection with *S*. *haematobium* and those variants identified as risk and protective alleles.

This study focusses on an underrepresented group among genetic association studies, as most population level genetic research focusses on individuals of European ancestry [24]. In addition, the genetic analysis of infectious disease susceptibility is an underdeveloped field relative to other diseases including metabolic diseases and cancer [110]. As such, this study provides novel analyses and findings on both an underrepresented population and disease. Genetic studies of individuals of African ancestry are particularly important in infectious diseases given the plethora of endemic diseases found on the continent, and the unique genetic background against which these occur. Population genetics is becoming increasingly recognised as an important modulator of infectious diseases, influencing susceptibility and disease severity [24]. Expanding analysis of the genetic basis of infectious disease susceptibility beyond populations of European ancestry is beneficial to understanding how such diseases differentially affect populations of different heritage and how interventions can be best informed to account for this. For example, the SNP rs12979680 in the *IL28B* gene encoding type III IFN-λ-3 has been found to associate with improved clearance and response to treatment in hepatitis C virus infection; however, this polymorphism is vastly more common among individuals of Asian and European ancestry compared to those of African ancestry, and this difference in host genetics is thought to partially underlie disparities in hepatitis C virus infection outcomes between African-Americans and European descendants [111,112]. Furthermore, genetics has been suggested as an underlying factor in the higher frequencies of allergic and atopic diseases observed among individuals of African heritage compared to those of European heritage [113,114]. Host genetics has also been hypothesised to be an underlying factor in the way in which the SARS-CoV-2 pandemic has manifested in sub-Saharan Africa, as substantially lower morbidity and mortality arising from the pandemic has occurred compared to European and North American countries [115,116]. The contextualisation of genetic associations with population-level allele frequencies is of additional benefit, as here in this study the novel protective and risk alleles were found to have higher and lower frequencies, respectively, in the study population relative to European populations. The frequencies of a range of immune system polymorphisms reported in this study is valuable as a contribution to the overall understanding of immunogenetics of both Zimbabweans and individuals of sub-Saharan African descent. Such understanding and continued research may contribute in the future to the use of population immunogenetics in the design and implementation of interventions against a range of infectious diseases.

The study presented here benefits from a number of strengths; this paper focusses on an underrepresented population and disease, thereby filling a gap in research into host genetic susceptibility. Furthermore, the sample size allowed the analysis of rarer alleles and the characterisation of the frequency of these SNPs within the population. However, there remains a number of limitations to the work described here. The exclusion of individuals with either *Plasmodium*, HIV or STH infection will, inevitably, have excluded a significant number of individuals and a particular demographic from participation. Some estimates have suggested that the prevalence of co-infection with *Plasmodium* and *Schistosoma* in some regions of sub-Saharan Africa may be as high as 30% [117], however the exclusion of co-infections from this study was necessary in order to control for potentially confounding concurrent immunological responses to *Plasmodium* infection. Additionally, although urinary schistosomiasis increases HIV risk and therefore may represent a significant proportion of all schistosome-infected individuals [118], prospective participants found to be infected with HIV were excluded to remove the confounding effects of the immunosuppressive nature of HIV infection. An additional limitation of this study is the unequal age distribution, in that the median age skews significantly young. While age was adjusted for in statistical modelling, it remains to be seen whether adult age-related effects exist within the results described here.

In summary, here we report on the frequency of SNPs within cytokine and TF genes and describe differences between the Zimbabwean study sample and African and European populations. In addition, we identify novel dominant protective and risk alleles at *IL4* rs2070874*T and *IFNG* rs2069727*G, respectively, for urogenital schistosomiasis and significantly associate the *IFNG* gene with schistosomiasis susceptibility for the first time. These findings add to the growing understanding of the role of genetic variation in schistosomiasis, emphasise the duality of T_H_ responses against schistosomes, and indicate important points of future investigation that may reveal more about the mechanisms of the host immune response to schistosome infection. These findings identify where genetic elements associated with elevated T_H_2 reactivity are more frequently observed among the study sample, contributing to a developing understanding of immunogenetics among individuals of African ancestry and highlight the need to improve the understanding of population-specific immunogenetics in the context of schistosomiasis, helminth infections, and neglected tropical diseases more widely.

## Supporting information

S1 Appendix Local *Schistosoma haematobium* Epidemiology. The local prevalence (%) and infection intensity (eggs/10ml urine) levels among participants, stratified by age group, sex and village.
(DOCX)

S2 Appendix Population Genotype Frequencies. The frequency of each genotype for each SNP under investigation among the study population.
(DOCX)

S3 Appendix SNP minor allele frequencies (MAFs) among Zimbabweans, Africans, and Europeans. Heatmap visualisation of the frequency of minor alleles of SNPs among three populations.
(DOCX)

S4 Appendix Linkage Disequilibrium Analysis. Full statistics from the linkage analysis performed on SNPs under investigation.
(DOCX)

S5 Appendix Principal Component Analysis Variance and Loading Scores. Full statistics from the principal component analysis performed on SNPs, including component variances and SNP loading scores.
(DOCX)

S6 Appendix PCA-Based Logistic and Linear Regression of Schistosome Infection and Infection Intensity. Plots and output tables from regression analysis of SNP principal components and schistosome infection.
(DOCX)
