# Supplementary material for: Frequency distribution of cytokine and associated transcription factor single nucleotide polymorphisms in Zimbabweans: Impact on schistosome infection and cytokine levels
Source: PLoS Negl Trop Dis. 2022 Jun 27;16(6):e0010536. doi: 10.1371/journal.pntd.0010536 (PMC9236240; doi:10.1371/journal.pntd.0010536)
Supplement: S1 Appendix — The local prevalence (%) and infection intensity (eggs/10ml urine) levels among participants, stratified by age group, sex and village. (DOCX) [file pntd.0010536.s001.docx]

**S1 Appendix. Local *Schistosoma haematobium* Epidemiology.** The local prevalence (%) and infection intensity (eggs/10ml urine) levels among participants, stratified by age group, sex and village.

|  | **N** | ***S. haematobium* Prevalence (%)** | **Infection Intensity (Eggs/10ml Urine)** | |
| --- | --- | --- | --- | --- |
|  |  |  | **Mean** | **SD** |
| **Total** | 827 | 44.498 | 29.033 | 100.646 |
| **Age (Years)** |  |  |  |  |
| 0-5 | 52 | 30.769 | 7.936 | 22.888 |
| 6-10 | 272 | 44.485 | 34.163 | 113.967 |
| 11-15 | 310 | 58.065 | 39.691 | 111.728 |
| 16-20 | 58 | 53.448 | 29.796 | 117.760 |
| 21-25 | 12 | 25.000 | 5.417 | 15.089 |
| 26-30 | 13 | 15.385 | 0.885 | 2.347 |
| >30 | 106 | 11.321 | 1.156 | 5.135 |
| **Sex** |  |  |  |  |
| Male | 364 | 51.648 | 42.757 | 124.862 |
| Female | 462 | 38.745 | 18.162 | 74.865 |
| **Village** |  |  |  |  |
| Magaya | 411 | 51.338 | 25.932 | 82.628 |
| Chipinda | 416 | 37.740 | 32.098 | 115.747 |
