# Supplementary material for: Frequency distribution of cytokine and associated transcription factor single nucleotide polymorphisms in Zimbabweans: Impact on schistosome infection and cytokine levels
Source: PLoS Negl Trop Dis. 2022 Jun 27;16(6):e0010536. doi: 10.1371/journal.pntd.0010536 (PMC9236240; doi:10.1371/journal.pntd.0010536)
Supplement: S2 Appendix — The frequency of each genotype for each SNP under investigation among the study population. (DOCX) [file pntd.0010536.s002.docx]

**S2 Appendix. Population Genotype Frequencies.** The frequency of each genotype for each SNP under investigation among the study population.

| **Chromo-some** | **Gene** | **Nucleotide Position** | **SNP ID** | **N** | **Ref. Allele** | **Minor Allele** | **Genotype** | **N (Frequency)** |
| --- | --- | --- | --- | --- | --- | --- | --- | --- |
| 1 | *IL10* | 206768519 | rs3024496 | 836 | T | C | T:T | 243 (0.291) |
|  |  |  |  |  |  |  | T:C | 414 (0.495) |
|  |  |  |  |  |  |  | C:C | 179 (0.214) |
|  |  | 206773062 | rs1800872 | 830 | A | C | A:A | 129 (0.155) |
|  |  |  |  |  |  |  | A:C | 396 (0.477) |
|  |  |  |  |  |  |  | C:C | 305 (0.367) |
|  |  | 206773552 | rs1800896 | 831 | A | G | A:A | 403 (0.485) |
|  |  |  |  |  |  |  | A:G | 347 (0.408) |
|  |  |  |  |  |  |  | G:G | 81 (0.095) |
| 2 | *STAT4* | 191032814 | rs925847 | 829 | C | T | C:C | 239 (0.288) |
|  |  |  |  |  |  |  | C:T | 427 (0.515) |
|  |  |  |  |  |  |  | T:T | 163 (0.197) |
|  |  | 191099907 | rs7574865 | 826 | T | G | T:T | 23 (0.028) |
|  |  |  |  |  |  |  | T:G | 165 (0.2) |
|  |  |  |  |  |  |  | G:G | 638 (0.751) |
|  |  | 191105394 | rs7582694 | 830 | C | G | C:C | 40 (0.055) |
|  |  |  |  |  |  |  | C:G | 262 (0.316) |
|  |  |  |  |  |  |  | G:G | 522 (0.629) |
| 5 | *IL13* | 132656717 | rs1881457 | 827 | A | C | A:A | 470 (0.568) |
|  |  |  |  |  |  |  | A:C | 308 (0.372) |
|  |  |  |  |  |  |  | C:C | 49 (0.059) |
|  |  | 132660151 | rs1295686 | 814 | A | G | A:A | 457 (0.561) |
|  |  |  |  |  |  |  | A:G | 299 (0.367) |
|  |  |  |  |  |  |  | G:G | 58 (0.071) |
|  |  | 132660272 | rs20541 | 825 | A | G | A:A | 45 (0.055) |
|  |  |  |  |  |  |  | A:G | 298 (0.361) |
|  |  |  |  |  |  |  | G:G | 482 (0.584) |
|  |  | 132660808 | rs848 | 824 | T | G | T:T | 250 (0.303) |
|  |  |  |  |  |  |  | T:G | 386 (0.468) |
|  |  |  |  |  |  |  | G:G | 188 (0.228) |
|  | *IL4* | 132672952 | rs2243248 | 832 | T | G | T:T | 527 (0.633) |
|  |  |  |  |  |  |  | T:G | 273 (0.328) |
|  |  |  |  |  |  |  | G:G | 32 (0.038) |
|  |  | 132673462 | rs2243250 | 810 | C | T | C:C | 49 (0.06) |
|  |  |  |  |  |  |  | C:T | 272 (0.336) |
|  |  |  |  |  |  |  | T:T | 489 (0.604) |
|  |  | 132674018 | rs2070874 | 825 | C | T | C:C | 175 (0.212) |
|  |  |  |  |  |  |  | C:T | 406 (0.492) |
|  |  |  |  |  |  |  | T:T | 244 (0.296) |
| 9 | *IL33* | 6213387 | rs928413 | 776 | G | A | G:G | 305 (0.393) |
|  |  |  |  |  |  |  | G:A | 332 (0.428) |
|  |  |  |  |  |  |  | A:A | 139 (0.179) |
|  |  | 6231239 | rs12551256 | 842 | A | G | A:A | 656 (0.779) |
|  |  |  |  |  |  |  | A:G | 175 (0.208) |
|  |  |  |  |  |  |  | G:G | 11 (0.013) |
|  |  | 6240084 | rs7025417 | 834 | T | C | T:T | 557 (0.668) |
|  |  |  |  |  |  |  | T:C | 250 (0.3) |
|  |  |  |  |  |  |  | C:C | 27 (0.032) |
| 10 | *GATA3* | 8047173 | rs4143094 | 816 | T | G | T:T | 190 (0.233) |
|  |  |  |  |  |  |  | T:G | 427 (0.523) |
|  |  |  |  |  |  |  | G:G | 199 (0.244) |
|  |  | 8060309 | rs3802604 | 820 | G | A | G:G | 497 (0.606) |
|  |  |  |  |  |  |  | G:A | 288 (0.351) |
|  |  |  |  |  |  |  | A:A | 35 (0.043) |
|  |  | 8074635 | rs1058240 | 831 | G | A | G:G | 28 (0.034) |
|  |  |  |  |  |  |  | G:A | 247 (0.297) |
|  |  |  |  |  |  |  | A:A | 556 (0.669) |
| 12 | *STAT6* | 68154443 | rs324015 | 816 | A | G | A:A | 28 (0.034) |
|  |  |  |  |  |  |  | A:G | 245 (0.3) |
|  |  |  |  |  |  |  | G:G | 543 (0.665) |
|  |  | 68156382 | rs11172106 | 823 | C | G | C:C | 375 (0.456) |
|  |  |  |  |  |  |  | C:G | 373 (0.453) |
|  |  |  |  |  |  |  | G:G | 75 (0.091) |
|  | *IFNG* | 68161231 | rs2069727 | 831 | A | G | A:A | 593 (0.714) |
|  |  |  |  |  |  |  | A:G | 222 (0.267) |
|  |  |  |  |  |  |  | G:G | 16 (0.019) |
|  |  | 57096317 | rs2069718 | 819 | A | G | A:A | 331 (0.404) |
|  |  |  |  |  |  |  | A:G | 385 (0.47) |
|  |  |  |  |  |  |  | G:G | 103 (0.126) |
|  |  | 57119092 | rs2069705 | 827 | C | T | C:C | 164 (0.198) |
|  |  |  |  |  |  |  | C:T | 430 (0.52) |
|  |  |  |  |  |  |  | T:T | 233 (0.282) |
| 17 | *STAT5B* | 42223863 | rs9900213 | 829 | G | T | G:G | 112 (0.135) |
|  |  |  |  |  |  |  | G:T | 334 (0.403) |
|  |  |  |  |  |  |  | T:T | 383 (0.462) |
|  |  | 42246955 | rs8082391 | 831 | C | A | C:C | 121 (0.146) |
|  |  |  |  |  |  |  | C:A | 403 (0.485) |
|  |  |  |  |  |  |  | A:A | 307 (0.369) |
|  | *STAT5A* | 42294404 | rs16967637 | 823 | C | A | C:C | 316 (0.384) |
|  |  |  |  |  |  |  | C:A | 395 (0.48) |
|  |  |  |  |  |  |  | A:A | 112 (0.136) |
|  |  | 42295383 | rs7217728 | 826 | T | C | T:T | 71 (0.086) |
|  |  |  |  |  |  |  | T:C | 349 (0.423) |
|  |  |  |  |  |  |  | C:C | 406 (0.492) |
|  |  | 42307544 | rs2272087 | 828 | T | C | T:T | 285 (0.344) |
|  |  |  |  |  |  |  | T:C | 434 (0.524) |
|  |  |  |  |  |  |  | C:C | 109 (0.132) |
|  | *TBX21* | 47731462 | rs4794067 | 814 | T | C | T:T | 594 (0.73) |
|  |  |  |  |  |  |  | T:C | 201 (0.247) |
|  |  |  |  |  |  |  | C:C | 19 (0.023) |
|  |  | 47743357 | rs11079788 | 835 | C | T | C:C | 794 (0.951) |
|  |  |  |  |  |  |  | C:T | 1 (0.001) |
|  |  |  |  |  |  |  | T:T | 40 (0.048) |
|  |  | 47748134 | rs16947078 | 822 | A | G | A:A | 579 (0.704) |
|  |  |  |  |  |  |  | A:G | 221 (0.269) |
|  |  |  |  |  |  |  | G:G | 22 (0.027) |
| X | *FOXP3* | 49249149 | rs2294021 | 823 | T | C | T:T | 487 (0.592) |
|  |  |  |  |  |  |  | T:C | 198 (0.241) |
|  |  |  |  |  |  |  | C:C | 138 (0.168) |
|  |  | 49259429 | rs2232365 | 826 | A | G | A:A | 124 (0.15) |
|  |  |  |  |  |  |  | A:G | 163 (0.197) |
|  |  |  |  |  |  |  | G:G | 539 (0.653) |
|  |  | 49265564 | rs11091253 | 834 | C | T | C:C | 586 (0.703) |
|  |  |  |  |  |  |  | C:T | 167 (0.2) |
|  |  |  |  |  |  |  | T:T | 81 (0.097) |
