# Supplementary material for: Frequency distribution of cytokine and associated transcription factor single nucleotide polymorphisms in Zimbabweans: Impact on schistosome infection and cytokine levels
Source: PLoS Negl Trop Dis. 2022 Jun 27;16(6):e0010536. doi: 10.1371/journal.pntd.0010536 (PMC9236240; doi:10.1371/journal.pntd.0010536)
Supplement: S3 Appendix — Heatmap visualisation of the frequency of minor alleles of SNPs among three populations. (DOCX) [file pntd.0010536.s003.docx]

**S3 Appendix. SNP minor allele frequencies (MAFs) among Zimbabweans, Africans, and Europeans.** Heatmap visualisation of the frequency of minor alleles of SNPs among three populations.

**
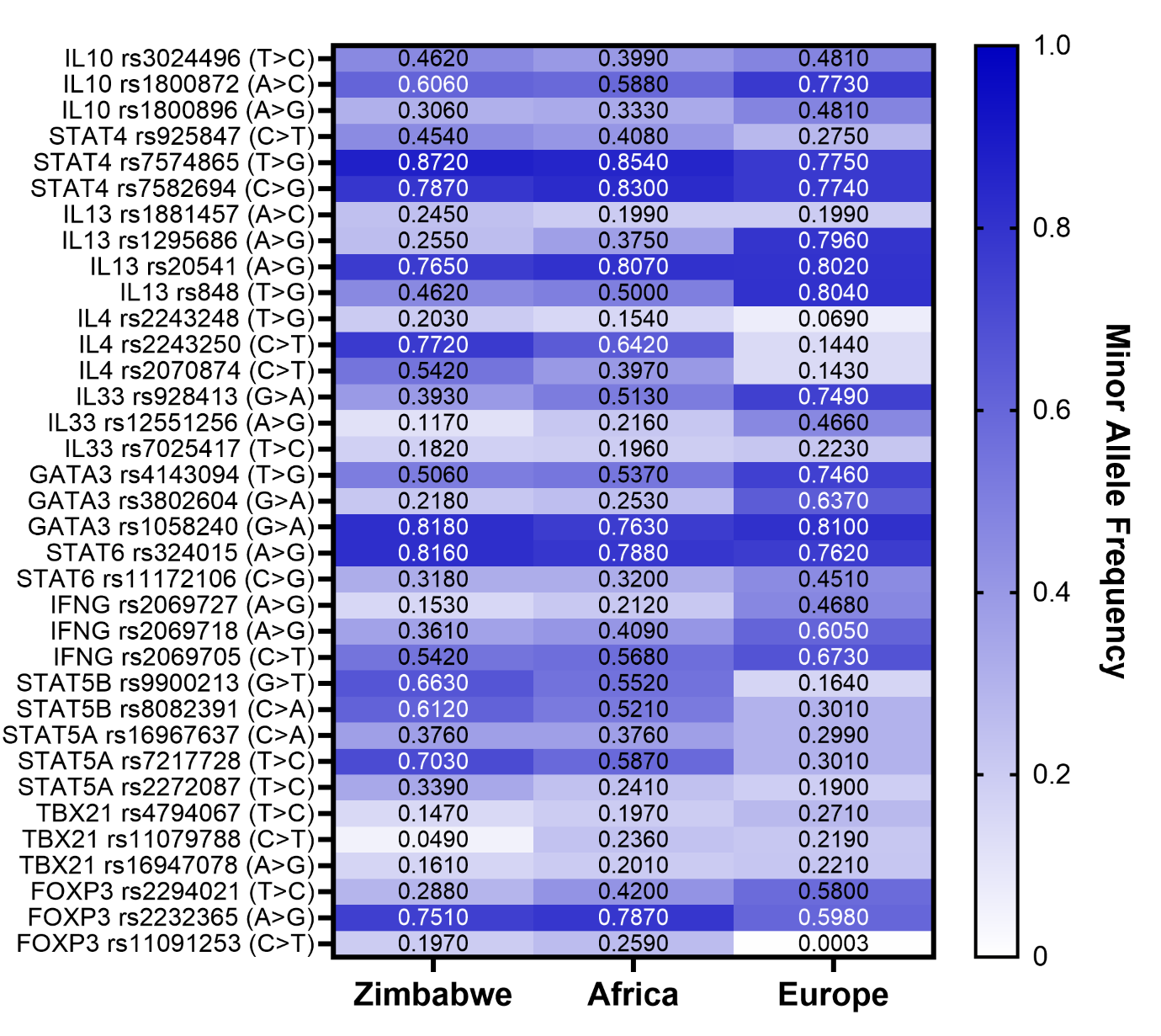
**

SNP minor allele frequencies (MAFs) among Zimbabweans, Africans, and Europeans. MAFs were more similar between Africans and Zimbabweans than between Europeans and Zimbabweans. In total, 23/35 (65.71%) of SNP MAFs were significantly different between the Zimbabwean sample and aggregated African populations, and 32/35 (91.43%) of SNP MAFs were significantly different between the Zimbabwean sample and aggregated European populations.
