# Supplementary material for: Frequency distribution of cytokine and associated transcription factor single nucleotide polymorphisms in Zimbabweans: Impact on schistosome infection and cytokine levels
Source: PLoS Negl Trop Dis. 2022 Jun 27;16(6):e0010536. doi: 10.1371/journal.pntd.0010536 (PMC9236240; doi:10.1371/journal.pntd.0010536)
Supplement: S4 Appendix — Full statistics from the linkage analysis performed on SNPs under investigation. (DOCX) [file pntd.0010536.s004.docx]

**S4 Appendix. Linkage Disequilibrium Analysis.** Full statistics from the linkage analysis performed on SNPs under investigation.

Chromosome 1

| **SNP 1 ID** | **SNP 2 ID** | **D’ (95% CIs)** | **r^2^** | **Distance (bp)** |
| --- | --- | --- | --- | --- |
| rs3024496 | rs1800872 | 0.973 (0.94-0.99) | 0.547 | 4543 |
| rs3024496 | rs1800896 | 0.786 (0.73-0.84) | 0.319 | 5033 |
| rs1800872 | rs1800896 | 0.952 (0.9-0.98) | 0.271 | 490 |

Chromosome 5

| **SNP 1 ID** | **SNP 2 ID** | **D’ (95% CIs)** | **r^2^** | **Distance (bp)** |
| --- | --- | --- | --- | --- |
| rs1881457 | rs1295686 | 0.227 (0.16-0.29) | 0.05 | 3434 |
| rs1881457 | rs20541 | 0.016 (-0.01-0.09) | 0.0 | 3555 |
| rs1881457 | rs848 | 0.11 (0.02-0.21) | 0.004 | 4091 |
| rs1881457 | rs2243248 | 0.039 (-0.01-0.12) | 0.001 | 16235 |
| rs1881457 | rs2070874 | 0.073 (0.0-0.19) | 0.002 | 17301 |
| rs1295686 | rs20541 | 0.93 (0.84-0.98) | 0.097 | 121 |
| rs1295686 | rs848 | 0.644 (0.56-0.71) | 0.158 | 657 |
| rs1295686 | rs2243248 | 0.108 (0.04-0.18) | 0.009 | 12801 |
| rs1295686 | rs2070874 | 0.244 (0.13-0.35) | 0.018 | 13867 |
| rs20541 | rs848 | 0.551 (0.45-0.64) | 0.09 | 536 |
| rs20541 | rs2243248 | 0.698 (0.53-0.81) | 0.042 | 12680 |
| rs20541 | rs2070874 | 0.345 (0.24-0.44) | 0.038 | 13746 |
| rs848 | rs2243248 | 0.2 (0.09-0.31) | 0.012 | 12144 |
| rs848 | rs2070874 | 0.08 (0.02-0.14) | 0.006 | 13210 |
| rs2243248 | rs2070874 | 0.295 (0.17-0.41) | 0.024 | 1066 |

Chromosome 9

| **SNP 1 ID** | **SNP 2 ID** | **D’ (95% CIs)** | **r^2^** | **Distance (bp)** |
| --- | --- | --- | --- | --- |
| rs12551256 | rs7025417 | 1.0 (0.75-1.0) | 0.028 | 8845 |

Chromosome 10

| **SNP 1 ID** | **SNP 2 ID** | **D’ (95% CIs)** | **r^2^** | **Distance (bp)** |
| --- | --- | --- | --- | --- |
| rs4143094 | rs3802604 | 0.864 (0.79-0.92) | 0.212 | 13136 |
| rs4143094 | rs1058240 | 0.354 (0.23-0.46) | 0.029 | 27462 |
| rs3802604 | rs1058420 | 0.738 (0.57-0.85) | 0.036 | 14326 |

Chromosome 12

| **SNP 1 ID** | **SNP 2 ID** | **D’ (95% CIs)** | **r^2^** | **Distance (bp)** |
| --- | --- | --- | --- | --- |
| rs2069727 | rs2069718 | 0.968 (0.92-0.99) | 0.307 | 1939 |
| rs2069727 | rs2069705 | 0.971 (0.9-1.0) | 0.151 | 6788 |
| rs2069718 | rs2069705 | 0.969 (0.93-0.99) | 0.458 | 4849 |

Chromosome 17

| **SNP 1 ID** | **SNP 2 ID** | **D’ (95% CIs)** | **r^2^** | **Distance (bp)** |
| --- | --- | --- | --- | --- |
| rs8082391 | rs16967637 | 0.326 (0.23-0.42) | 0.042 | 47449 |
| rs8082391 | rs7217728 | 0.902 (0.86-0.94) | 0.559 | 48428 |
| rs8082391 | rs2272087 | 0.156 (0.05-0.26) | 0.01 | 60589 |
| rs16967367 | rs7217728 | 0.929 (0.87-0.97) | 0.232 | 979 |
| rs16967367 | rs2272087 | 0.063 (0.0-0.14) | 0.004 | 13140 |
| rs7217728 | rs2272087 | 0.265 (0.15-0.37) | 0.02 | 12161 |
| rs4794067 | rs16947078 | 0.414 (0.34-0.48) | 0.152 | 16672 |

X Chromosome

| **SNP 1 ID** | **SNP 2 ID** | **D’ (95% CIs)** | **r^2^** | **Distance (bp)** |
| --- | --- | --- | --- | --- |
| rs2294021 | rs2232365 | 0.971 (0.9-1.0) | 0.132 | 10280 |
| rs2294021 | rs11091253 | 0.935 (0.89-0.97) | 0.558 | 16415 |
| rs2232365 | rs11091253 | 1.0 (0.93-1.0) | 0.089 | 6135 |
