# Supplementary material for: Frequency distribution of cytokine and associated transcription factor single nucleotide polymorphisms in Zimbabweans: Impact on schistosome infection and cytokine levels
Source: PLoS Negl Trop Dis. 2022 Jun 27;16(6):e0010536. doi: 10.1371/journal.pntd.0010536 (PMC9236240; doi:10.1371/journal.pntd.0010536)
Supplement: S5 Appendix — Full statistics from the principal component analysis performed on SNPs, including component variances and SNP loading scores. (DOCX) [file pntd.0010536.s005.docx]

**S5 Appendix. Principal Component Analysis Variance and Loading Scores.** Full statistics from the principal component analysis performed on SNPs, including component variances and SNP loading scores.

**Table 1: Principal component % variance.**

| Component | % of Variance | Cumulative % |
| --- | --- | --- |
| 1 | 7.399 | 7.399 |
| 2 | 6.685 | 14.083 |
| 3 | 6.468 | 20.552 |
| 4 | 5.985 | 26.537 |
| 5 | 5.607 | 32.144 |
| 6 | 5.380 | 37.524 |
| 7 | 4.895 | 42.419 |
| 8 | 4.525 | 46.944 |
| 9 | 4.219 | 51.164 |
| 10 | 3.695 | 54.859 |
| 11 | 3.361 | 58.219 |
| 12 | 3.194 | 61.414 |
| 13 | 3.154 | 64.567 |
| 14 | 2.952 | 67.520 |

**Table 2: Factor loadings of principal components.**

| **SNP ID** | **Gene** | **Component** | | | | | | | | | | | | | |
| --- | --- | --- | --- | --- | --- | --- | --- | --- | --- | --- | --- | --- | --- | --- | --- |
|  |  | **1** | **2** | **3** | **4** | **5** | **6** | **7** | **8** | **9** | **10** | **11** | **12** | **13** | **14** |
| rs7217728 | STAT5A | 0.934 |  |  |  |  |  |  |  |  |  |  |  |  |  |
| rs8082391 | STAT5B | 0.804 |  |  |  |  |  |  |  |  |  |  |  |  |  |
| rs9900213 | STAT5B | 0.723 |  |  |  |  |  |  |  |  |  |  |  |  |  |
| rs16967637 | STAT5A | 0.577 |  |  |  |  |  |  |  |  |  |  |  |  |  |
| rs3024496 | IL10 |  | 0.896 |  |  |  |  |  |  |  |  |  |  |  |  |
| rs1800872 | IL10 |  | 0.892 |  |  |  |  |  |  |  |  |  |  |  |  |
| rs1800896 | IL10 |  | 0.802 |  |  |  |  |  |  |  |  |  |  |  |  |
| rs2069718 | IFNG |  |  | 0.916 |  |  |  |  |  |  |  |  |  |  |  |
| rs2069705 | IFNG |  |  | 0.827 |  |  |  |  |  |  |  |  |  |  |  |
| rs2069727 | IFNG |  |  | 0.755 |  |  |  |  |  |  |  |  |  |  |  |
| rs2294021 | FOXP3 |  |  |  | 0.906 |  |  |  |  |  |  |  |  |  |  |
| rs11091253 | FOXP3 |  |  |  | 0.866 |  |  |  |  |  |  |  |  |  |  |
| rs2232365 | FOXP3 |  |  |  | 0.621 |  |  |  |  |  |  |  |  |  |  |
| rs11079788 | TBX21 |  |  |  |  | 0.802 |  |  |  |  |  |  |  |  |  |
| rs16947078 | TBX21 |  |  |  |  | 0.782 |  |  |  |  |  |  |  |  |  |
| rs4794067 | TBX21 |  |  |  |  | 0.764 |  |  |  |  |  |  |  |  |  |
| rs2070874 | IL4 |  |  |  |  |  | 0.876 |  |  |  |  |  |  |  |  |
| rs2243250 | IL4 |  |  |  |  |  | 0.842 |  |  |  |  |  |  |  |  |
| rs2243248 | IL4 |  |  |  |  |  | -0.461 |  |  |  |  |  |  |  |  |
| rs7582694 | STAT4 |  |  |  |  |  |  | 0.928 |  |  |  |  |  |  |  |
| rs7574865 | STAT4 |  |  |  |  |  |  | 0.925 |  |  |  |  |  |  |  |
| rs1295686 | IL13 |  |  |  |  |  |  |  | 0.778 |  |  |  |  |  |  |
| rs848 | IL13 |  |  |  |  |  |  |  | 0.721 |  |  |  |  |  |  |
| rs20541 | IL13 |  |  |  |  |  |  |  | 0.685 |  |  |  |  |  |  |
| rs3802604 | GATA3 |  |  |  |  |  |  |  |  | 0.82 |  |  |  |  |  |
| rs4143094 | GATA3 |  |  |  |  |  |  |  |  | 0.799 |  |  |  |  |  |
| rs1058240 | GATA3 |  |  |  |  |  |  |  |  | 0.503 |  |  |  |  |  |
| rs11172106 | STAT6 |  |  |  |  |  |  |  |  |  | 0.797 |  |  |  |  |
| rs324015 | STAT6 |  |  |  |  |  |  |  |  |  | 0.789 |  |  |  |  |
| rs12551256 | IL33 |  |  |  |  |  |  |  |  |  |  | 0.791 |  |  |  |
| rs7025417 | IL33 |  |  |  |  |  |  |  |  |  |  | -0.588 |  | 0.519 |  |
| rs1881457 | IL13 |  |  |  |  |  |  |  |  |  |  |  | 0.848 |  |  |
| rs928413 | IL33 |  |  |  |  |  |  |  |  |  |  |  |  | 0.874 |  |
| rs2272087 | STAT5A |  |  |  |  |  |  |  |  |  |  |  |  |  | 0.647 |
| rs925847 | STAT4 |  |  |  |  |  |  |  |  |  |  |  |  |  | 0.604 |
