# Supplementary material for: Frequency distribution of cytokine and associated transcription factor single nucleotide polymorphisms in Zimbabweans: Impact on schistosome infection and cytokine levels
Source: PLoS Negl Trop Dis. 2022 Jun 27;16(6):e0010536. doi: 10.1371/journal.pntd.0010536 (PMC9236240; doi:10.1371/journal.pntd.0010536)
Supplement: S6 Appendix — Plots and output tables from regression analysis of SNP principal components and schistosome infection. (DOCX) [file pntd.0010536.s006.docx]

***S6 Appendix. PCA-Based Logistic and Linear Regression of Schistosome Infection and Infection Intensity.*** *Plots and output tables from regression analysis of SNP principal components and schistosome infection.*





***A) Logistic regression of egg positivity: ORs for each principal component****. ORs are per 1 unit increase in component score. This analysis identified that PC3, representing IFNG SNPs, is associated with a significant OR of 1.257 (95% CIs: 1.054-1.499, p = 0.011). PC4, representing FOXP3 SNPs, was associated with a significant OR of 0.803 (95% CIs: 0.672-0.96, p = 0.016). PC6, representing IL4 SNPs, was associated with a significant OR of 0.733 (95% CIs: 0.61-0.88, p = 0.00088).* ***B) Multiple linear regression of infection intensity: β coefficients for each principal component****. β coefficients are per 1 unit increase in component score. This analysis identified that PC6, representing IL4 SNPs, was significantly and negatively associated with infection intensity (β = -0.111, 95% CIs: -0.19 - -0.036, p = 0.0063). Logistic and multiple linear regression models are adjusted for village, sex, and age group. OR = odds ratio, CI = confidence interval.*

***Logistic Regression Output Table***

|  | B | Wald | Exp(B) (95% CIs) | p-value |
| --- | --- | --- | --- | --- |
| Sex | -0.2969 | 0.1826 | 0.7431 (0.5196-1.0629) | 0.1 |
| Age | -0.2633 | 0.0595 | 0.7685 (0.6839-0.8636) | <0.0001 |
| Village | -0.7475 | 0.1813 | 0.4735 (0.3319-0.6756) | <0.0001 |
| PC1 | 0.0122 | 0.0896 | 1.0122 (0.8493-1.2065) | 0.89 |
| PC2 | -0.066 | 0.0877 | 0.9362 (0.7883-1.1118) | 0.45 |
| PC3 | 0.0852 | 0.0871 | 1.0889 (0.918-1.2916) | 0.33 |
| PC4 | -0.1282 | 0.0887 | 0.8796 (0.7393-1.0466) | 0.15 |
| PC5 | -0.0754 | 0.0935 | 0.9274 (0.772-1.114) | 0.42 |
| **PC6** | **-0.22** | **0.0911** | **0.8026 (0.6713-0.9595)** | **0.016** |
| PC7 | -0.0432 | 0.0928 | 0.9577 (0.7985-1.1487) | 0.64 |
| PC8 | 0.0456 | 0.0883 | 1.0467 (0.8803-1.2445) | 0.61 |
| PC9 | -0.1018 | 0.0896 | 0.9032 (0.7577-1.0766) | 0.26 |
| PC10 | -0.0408 | 0.089 | 0.96 (0.8064-1.1429) | 0.65 |
| PC11 | 0.0853 | 0.0916 | 1.089 (0.9101-1.3031) | 0.35 |
| PC12 | -0.0596 | 0.0881 | 0.9422 (0.7928-1.1197) | 0.5 |
| PC13 | -0.0568 | 0.088 | 0.9448 (0.795-1.1227) | 0.52 |
| PC14 | 0.0451 | 0.0878 | 1.0461 (0.8808-1.2424) | 0.61 |
| Constant | 1.2129 | 0.2401 |  | <0.0001 |

***Linear Regression Output Table***

|  | B (95% CIs) | β (95% CIs) | p-value |
| --- | --- | --- | --- |
| Constant | 0.9539 (0.7964-1.1114) |  | <0.0001 |
| Age | -0.2057 (-0.3357- -0.0758) | -0.1328 (-0.2166- -0.04890 | 0.0020 |
| Sex | -0.068 (-0.1058- -0.0302) | -0.1501 (-0.2336- -0.0666) | 0.00040 |
| Village | -0.1227 (-0.2475-0.002) | -0.0798 (-0.1609-0.0013) | 0.054 |
| PC1 | -0.0324 (-0.0947-0.03) | -0.0414 (-0.121-0.0383) | 0.31 |
| PC2 | -0.0199 (-0.081-0.0412) | -0.026 (-0.1058-0.0538) | 0.52 |
| PC3 | 0.024 (-0.0365-0.0845) | 0.0317 (-0.0481-0.1115) | 0.44 |
| PC4 | -0.0564 (-0.1185-0.0056) | -0.0725 (-0.1522-0.0072) | 0.075 |
| PC5 | -0.0262 (-0.0904-0.038) | -0.0326 (-0.1127-0.0474) | 0.42 |
| PC6 | -0.0516 (-0.115-0.0.0119) | -0.0653 (-0.1455-0.015) | 0.11 |
| PC7 | -0.0011 (-0.065-0.0629) | -0.0013 (-0.0818-0.0791) | 0.97 |
| PC8 | -0.0119 (-0.0737-0.0449) | -0.0154 (-0.0952-0.0644) | 0.71 |
| PC9 | -0.0328 (-0.0949-0.0294) | -0.0421 (-0.1219-0.0377) | 0.30 |
| PC10 | -0.0163 (-0.0774-0.0449) | -0.0213 (-0.1011-0.0586) | 0.60 |
| PC11 | 0.0322 (-0.031-0.0954) | 0.0408 (-0.0392-0.1207) | 0.32 |
| PC12 | -0.021 (-0.0817-0.0397) | -0.0277 (-0.1078-0.0523) | 0.50 |
| PC13 | -0.018 (-0.0789-0.043) | -0.0235 (-0.1033-0.0563) | 0.56 |
| PC14 | -0.0254 (-0.0863-0.0354) | -0.0336 (-0.114-0.0468) | 0.41 |
